# Weaker and more frequent Mediterranean sea breezes in a warming climate

**DOI:** 10.1038/s41598-026-47025-4

**Published:** 2026-05-15

**Authors:** Shalenys Bedoya-Valestt, Cesar Azorin-Molina, Nuria P. Plaza-Martin, Lorenzo Minola, Luis Gimeno, Miguel Andres-Martin, Sergio M. Vicente-Serrano, Deliang Chen

**Affiliations:** 1 Centro de Investigaciones Sobre Desertificación, Consejo Superior de Investigaciones Científicas (CIDE, CSIC-UV-Generalitat Valenciana), Climate, Atmosphere and Ocean Laboratory (Climatoc-Lab), Ctra. CV-315 Km 10.7, 46113 Moncada, Valencia Spain; 2 Interuniversity Department of Regional and Urban Studies and Planning (DIST), Politecnico and University of Turin, Turin, Italy; 3 Centro de Investigación Mariña, Environmental Physics Laboratory (EPhysLab), Universidade de Vigo, Ourense, Spain; 4 Galicia Supercomputing Center (CESGA), Santiago de Compostela, Spain; 5 Instituto Pirenaico de Ecología, Consejo Superior de Investigaciones Científicas (IPE-CSIC), Zaragoza, Spain; 6 Ministry of Education Key Laboratory for Earth System Modeling, Department of Earth System Science, Tsinghua University, Beijing, 100084 China; 7 Institute for Carbon Neutrality, Tsinghua University, Beijing, 100084, China

**Keywords:** Sea breezes, Speeds, Occurrence, Mediterranean, Atmospheric circulation, Climate sciences, Environmental sciences

## Abstract

The Mediterranean, a climate change hotspot, has experienced accelerated warming, which is expected to influence sea breezes (SB) speed and occurrence. The extent to which these local winds have changed remains unclear due to the scarcity of multidecadal observations. Here we present a regional-scale assessment of SB across the Western Mediterranean, based on an unprecedented 41-year homogenized observational database spanning multiple countries and stations. Since the 1980s, SB have become weaker while more frequent. We link these changes to the interplay of thermodynamic and dynamic mechanisms driven by regional warming. The warming-induced weakening of SB speeds is further amplified during heatwaves, with speed reductions of up to 10%. In contrast, their winter occurrence has risen by roughly 10% per decade, consistent with a higher frequency of anticyclonic conditions. These findings offer a new understanding of SB response to the warming of the Mediterranean, with key implications for climate-related risks such as heat extremes and air quality.

**Supplementary Information:**

The online version contains supplementary material available at 10.1038/s41598-026-47025-4.

## Introduction

The Sixth Assessment Report of the Intergovernmental Panel on Climate Change (IPCC) indicates that the Mediterranean region has warmed by 1.5 °C above pre-industrial levels (relative to the 1850–1900 baseline), outpacing the global mean rate of 1.1 °C^1,2^. This accelerated warming encompasses sea surface temperature rising at ~ 0.4 °C decade^−1^^3,4^ and air temperature over land at ~ 0.5 °C decade^−1^^1^, corresponding to increases roughly 20% and 40% faster than the respective global averages^2,4^. This uneven warming is expected to enhance the land-sea thermal contrast^5–7^, which is a primary driver of local winds. Thus, coastal circulations such as sea breezes (SB) could be affected in their speeds and frequency.

Overall, global winds are undergoing significant changes in response to global warming and associated anthropogenic feedbacks^8^. Ocean surface winds have strengthened since the 1980s^9^, while coastal and terrestrial near-surface winds in the Northern Hemisphere weakened^9,10^ and, despite regional uncertainties^11^, they are projected to continue declining by the end of the century^12^. This phenomenon, termed global ‘stilling’^13^, raises the question of whether winds that are strongly driven by thermodynamic mechanisms, such as SB, exhibit a comparable decline, as their underlying mechanisms differ from those of winds associated with mesoscale-to-synoptic systems. However, a comprehensive understanding of SB changes under recent warming remains unclear, as high-resolution observational records over the past half-century are rarely available for multi-decadal assessments prior to the automation of weather stations in the 1990s–2000s^14^. As a result, most existing literature on SB climatology is constrained to short time spans, ranging from days to a few years^15–17^, and often rely on numerical simulations given their complex interaction with orography^18,19^.

Recently, long-term SB variability has been addressed using reanalysis products^19–21^ and the longest available records^15,22^. Owing to the limited ability of reanalysis to represent local winds, observations provide the most direct measure for assessing SB trends. Observational assessments, however, remain scarce and site-specific^15,23,24^. Reported SB changes reflect strong spatial heterogeneity^19,25–27^ rather than a systematic global pattern. For instance, weakened SB speeds have been observed along the coasts of Eastern Spain^25^ and China^21^, particularly over the main coastal megacities^23,24,28^. In contrast, they have strengthened in various tropical and subtropical cities in the Southern Hemisphere^27,29^. Regarding SB days, they have largely increased at most reported sites^25^, except in some densely urbanized cities where they declined (e.g., Shanghai^23^, Valencia^25^, Cairo^27^, Hong Kong^28^. Yet, observed SB trends at the regional scale remain unquantified (i.e., in terms of basin-wide rates of change), particularly in the Mediterranean basin, a recognized climate change hotspot^1,30^. A robust, multi-decadal regional assessment is strongly needed to fill this gap.

While site-specific trends highlight local physical controls, anthropogenic climate change is also driving changes in regional circulation that introduce an additional factor conditioning SB response to warming. These include ongoing shifts in mid-latitude high-pressure systems, e.g., the expansion of the Azores High in the twentieth century^31^, or increased anticyclonic blocking over the Mediterranean linked to a wavier jet stream^32,33^. In particular, a favored persistence of anticyclonic regimes over the western Mediterranean is characterized by enhanced subsidence and modified synoptic-scale winds. These changes weaken surface pressure gradients and enhance clear skies, favoring the radiative heating essential for the SB to develop. In addition, the strong subsidence and warm-air advection associated with high-pressure systems can strengthen low-level inversions, and thus constrain the vertical growth of the SB cell^34,35^, making it challenging to isolate the contribution of dynamical factors to local SB changes from thermodynamic forcing and intrinsic variability.

The implications of SB for society and the environment are wide-ranging. A better understanding of their changes is particularly essential under anthropogenic climate change, as they modulate the effects of a warmer Mediterranean on the region’s approximately 500 million inhabitants. With heatwaves becoming more frequent and severe^36^, SB play a key role in easing heat stress^37^. Yet, they can also recirculate pollutants along coastal and inland areas from days to weeks^38^, posing a risk to human health^39^. Furthermore, they fuel summer deep convection^40,41^, which is a major mechanism of severe winds and large hail in the region^42^, and can also influence moisture transport and evapotranspiration processes, which play a crucial role in determining drought severity^43^.

This work presents the first regional-scale assessment of SB changes across the Western Mediterranean basin based on an unprecedented 41-year observational database (1981–2021). We quantify regional (basin-wide) trends in SB speed and occurrence, moving beyond site-specific analyses and linking the observed changes to the recent warming of the Mediterranean and climate-driven cascading responses in atmospheric circulation feedbacks. Our findings provide a new understanding of how SB are responding to regional warming, which is essential for assessing climate risks and improving future projections.

## Results

### Sea breezes in change: 41 years of shifts over the Western Mediterranean basin

**Fig. 1:**
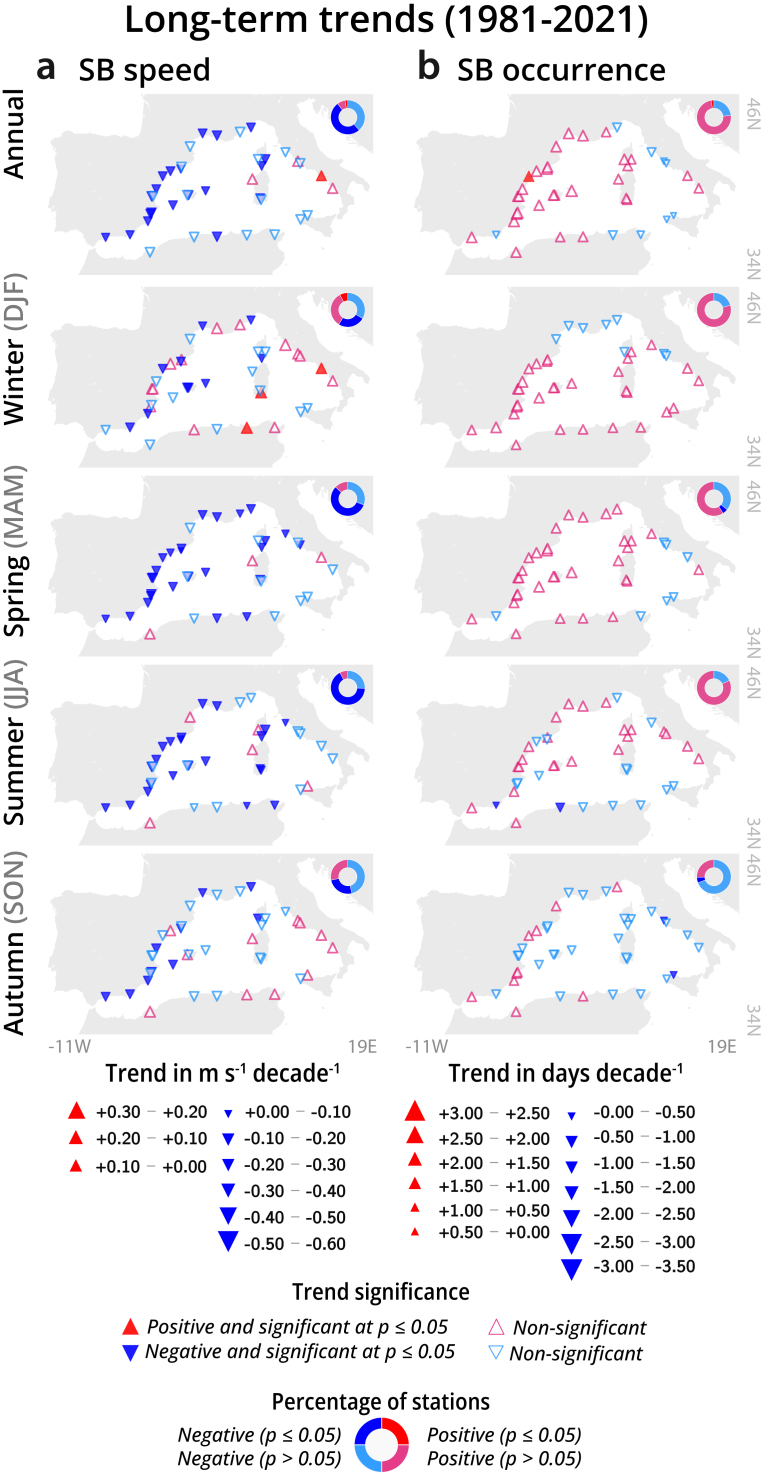
Changes in Mediterranean SB speed and occurrence for 1981–2021. Annual and seasonal spatial distribution of the sign, magnitude and statistical significance of trends for SB **a** speed (in m s^−1^ decade^−1^) and **b** occurrence (in days decade^−1^) across the Western Mediterranean Basin for 1981–2021. The circles contain the percentage of stations displaying positive and negative significant (*p* ≤ 0.05) and non-significant trends. Meteorological stations are shown in Fig. S1 and Table S1. The magnitude of the SB trends per station is given in Tables S4 and S5 in supplementary material. Maps were produced using the Python Matplotlib Basemap Toolkit (v1.3.6, https://matplotlib.org/basemap/), with coastlines and political boundaries from GSHHG^44^ and GMT^45^ contributors under the LGPL-3.0 license.

After identifying 41-years of SB episodes across the Western Mediterranean (see Methods), we investigated observed trends of SB speed and occurrence to show that Mediterranean SB have become weaker since 1981 (Fig. 1a), while the generalized spatial pattern points to more frequent SB across the region (Fig. 1b).

**Fig. 2:**
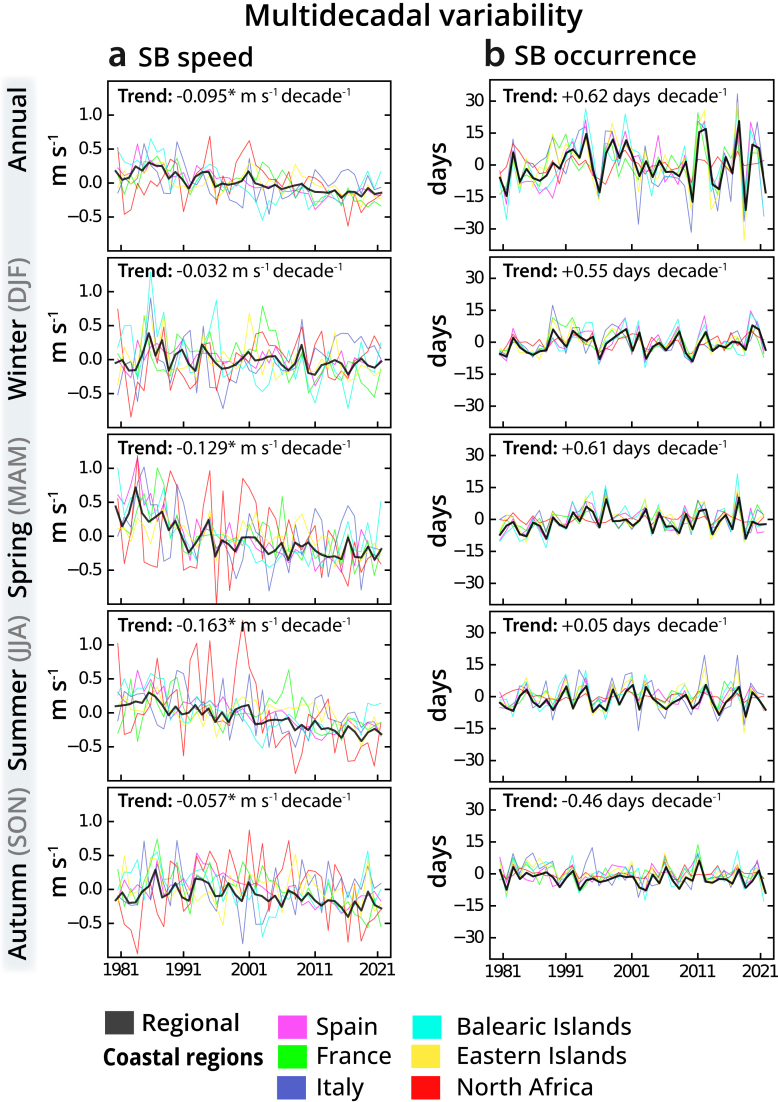
Regional and coastal multidecadal variability in SB anomalies. Time series of SB **a** speed and **b** occurrence at annual and seasonal scale. For each panel, the linear trend for the Western Mediterranean region is shown (black line). Statistically significant trends (*p* ≤ 0.05) are indicated by an asterisk (*).

The basin-wide assessment evidences a dominance of weaker SB at approximately 90% of the stations at almost all timescales for 1981–2021, being these decreases particularly significant and stronger in Spain and the Balearic Islands (Fig. 1a, Table S3). The SB speed reduction is more pronounced in spring-summer, with decreases of up to 10% per decade (*p* ≤ 0.05; Figs. 1a, S2a), while autumn and winter show increases of up to 12% per decade dominating up to 41% of the weather stations along the coasts of Italy, North Africa and Spain (Fig. 1a). Further, the spatial trends evidence a particular linear pattern in relation to the magnitude of change at each station (Fig. S2a): e.g., although decreasing speeds, the percentage of change at Eastern Islands is smaller than the change over Spain, France or Balearic Islands. However, despite the differences found at sparse stations, and the higher inter-annual variability evidenced by some coastal regions (e.g., North Africa), the time series shows the SB speed weakening to be persistent (Fig. 2a), while the multi-trend analysis across different temporal windows confirms the decline to be significant over the 41-years analyzed (Fig. S3a).

The spatial distribution of the SB occurrence reveals a general tendency toward increases at 77–80% of the stations on an annual basis, particularly during winter and spring, although these trends are statistically non-significant (Fig. 1b). The positive trends are notably greater over Spain and the Balearic Islands, with trends amounting to + 2 days per decade on average (i.e., 10% days per decade; Fig. 1b, S2b). Weaker reductions in the occurrence are seen along Italy (annually and in spring), France (in winter) and North Africa (in summer; Fig. 1b). In addition, non-significant declines prevail across the region in autumn (74% of the stations). Despite the strong multidecadal variability (Fig. 2b), spatial linear trends point to predominantly positive changes across most of the stations (Fig. S2b), while the multiyear-sized running windows highlight a marked increase in winter occurrence in recent decades (Fig. S3b).

### The role of a warmer Mediterranean on sea breeze trends

**Fig. 3:**
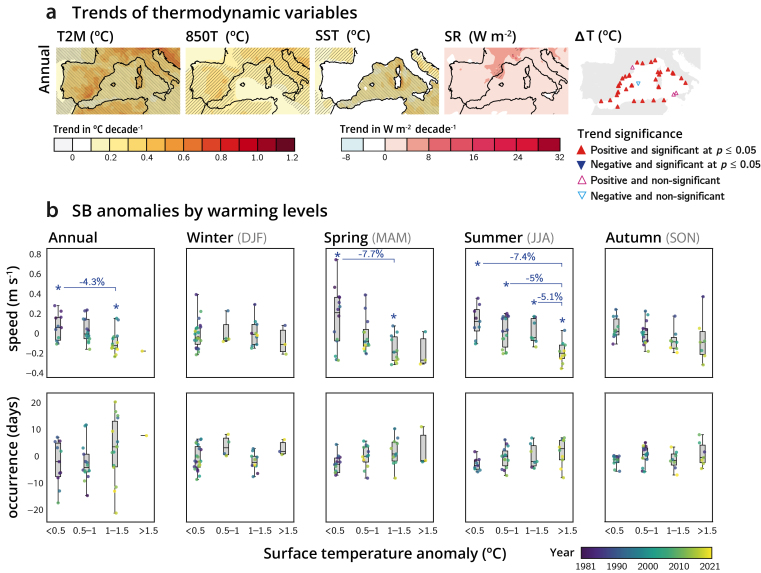
Annual changes in thermodynamic variables across the Western Mediterranean basin for 1981–2021, and the relationship of regional SB changes with temperature rise. **a** Spatial trends of surface air temperature (T2M), air temperature at 850 hPa (850T), sea surface temperature (SST), solar net radiation (SR) and the land-sea temperature contrast (∆T). Hatches indicate areas with statistically significant trends at *p* ≤ 0.05. **b** Relationship between anomalies in SB speed and occurrence, and surface temperature anomalies (relative to the 1951–1980 baseline) for 1981–2021. Statistically significant changes across surface temperature anomaly ranges are indicated in blue with an asterisk (*p* ≤ 0.05). Maps in **a** were produced using the Python Matplotlib Basemap Toolkit (v1.3.6, https://matplotlib.org/basemap/), with coastlines and political boundaries from GSHHG^44^ and GMT^45^ contributors under the LGPL-3.0 license.

**Fig. 4:**
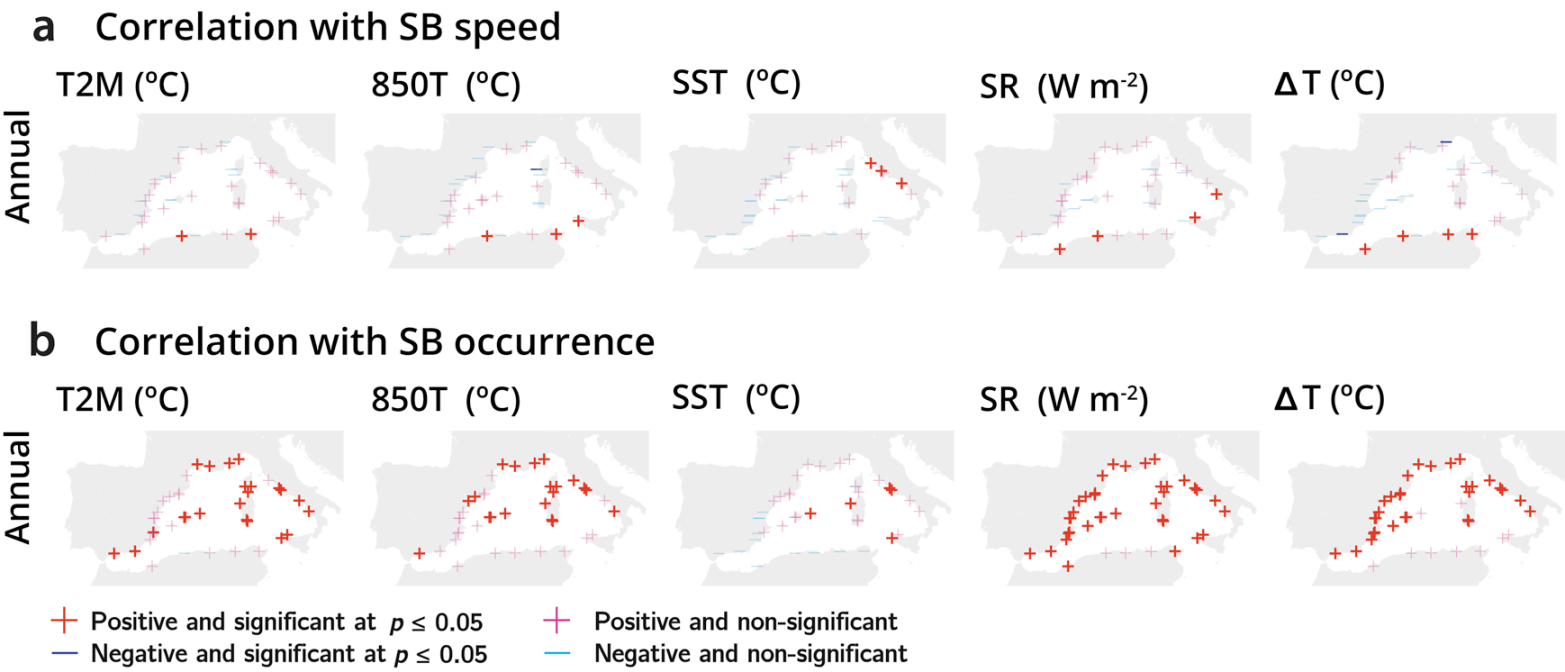
Spatial distribution of Pearson correlation coefficients between SB and thermodynamic variables. Pearson correlations between detrended anomalies of SB **a** speed and **b** occurrence and surface air temperature (T2M), air temperature at 850 hPa (850T), sea surface temperature (SST), net solar radiation (SR), and the land–sea temperature contrast (ΔT). Statistically significant correlations are highlighted (*p* ≤ 0.05). Seasonal correlations are shown in Fig. S5 in supplementary material. Maps were produced using the Python Matplotlib Basemap Toolkit (v1.3.6, https://matplotlib.org/basemap/), with coastlines and political boundaries from GSHHG^44^ and GMT^45^ contributors under the LGPL-3.0 license.

The Western Mediterranean has warmed significantly since 1981, with temperature increases of 1.3 °C at the surface (T2M, surface air temperature), 1.2 °C at low-levels (850T, air temperature at 850 hPa) and 1 °C at the sea surface (SST, sea surface temperature), together with a marked rise in solar radiation (SR) of 12.12 W m^−2^ (Fig. 3a). The faster warming over land relative to the sea has significantly strengthened the land-sea thermal contrast (∆T) across all timescales (Figs. 3a, S4), which is likely influencing changes in the SB occurrence (Figs. 3b and 4a and b). The response of SB to surface temperature rise is particularly striking: as warming increases up to 1.5 °C, SB speeds systematically weaken by 4–8%, especially in spring and summer (Fig. 3b). The SB occurrence shows a non-significant rise of up to 12%, most evident during the accelerated warming of 1–1.5 °C that has dominated since 2015, when regional warming became more pronounced.

Although basin-wide correlations between warming-related variables and detrended SB speed anomalies are generally weak and spatially heterogeneous (Fig. 4a), SB speed exhibit negative relationships with 850T, SST, and ∆T over Spain, particularly in spring and autumn, where correlations become significant (seasonal correlations in Fig. S5a). The unexpected contribution of intensified regional ∆T to the weakened SB speed suggest potential modifications in the vertical structure of the SB circulation due to thermodynamic changes. To further show this linear relationship, Fig. S6 shows that rising warming trends are associated with decreasing SB speed trends across most of the stations.

In contrast, Fig. 4b displays widespread positive and significant correlations between warming-related variables and the detrended anomalies of SB occurrence at all time-scales (seasonal correlations in Fig. S5b), suggesting that warming may contribute to increased SB occurrence in the western Mediterranean at over 60% of the weather stations (except North Africa). SR and ∆T are strongly associated with SB development in more than 90% of stations across the basin throughout the year, while the influence of T2M and 850T is more significant along the coasts of Spain, Italy and the Islands, prevailing in more than 64% of the stations. The results of the correlation analysis are consistent with the trend-on-trend analysis in Fig. S6, indicating that positive SB occurrence trends relate to rising warming trends across most of the stations.

**Fig. 5:**
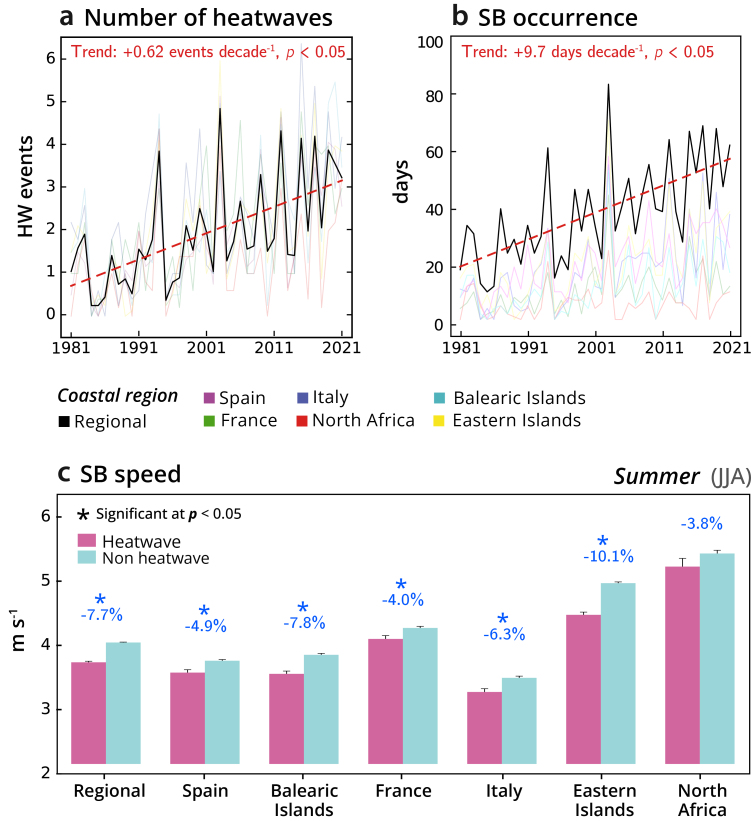
Summer heatwaves effect on SB occurrence and speed across the Western Mediterranean Basin for 1981–2021. Regional and coastal trends of **a** heatwave events, and **b** summer SB occurrence during heatwaves. **c** Differences in summer SB speed during heatwaves (HW) and non-heatwaves (NHW) episodes. Statistical significance at *p* ≤ 0.05 is reported with an asterisk (*).

The outcomes of the correlation analysis already suggest a link between regional warming and changes in SB, which we investigate further by focusing on heatwaves as an extreme event of warming. Our analyses reveal a significant increase in the frequency and duration of Mediterranean heatwaves since 1981, affecting more than 90% of the stations (Figs. 5 and S7). Crucially, as atmospheric heatwaves become more frequent, they are consistently accompanied by changes in SB, with SB occurrence increasing significantly during these events (Fig. 5b), a signal that becomes most robust at the regional scale when aggregating all coasts. At the same time, SB speed weaken systematically under heatwave conditions (Fig. 5c), with significant reductions averaging 8% at the regional scale and reaching up to 10% in the Eastern Islands, a decline pattern observed across all coasts and individual stations (Fig. S8). Beyond their statistical significance, these results provide observational evidence that extreme warming episodes are associated with increased SB occurrence but reduced speed, consistent with a potential influence of thermodynamic forcing on regional and seasonal SB changes.

### Link to changes in the atmospheric circulation

**Fig. 6:**
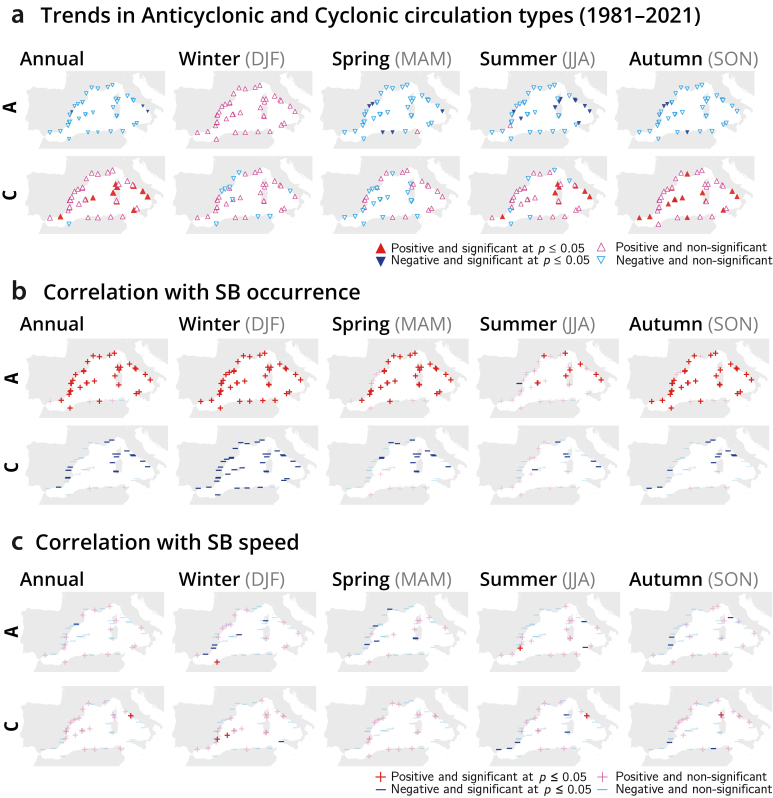
Anticyclonic and cyclonic circulation trends and their relationship with detrended SB anomalies. Annual and seasonal spatial distribution of the sign, magnitude and statistical significance of trends for JC **a** anticyclones (A) and cyclones (C), and their Pearson correlation with SB **b** occurrence and **c** speed. Statistically significant correlations are indicated (*p* ≤ 0.05). Maps were produced using the Python Matplotlib Basemap Toolkit (v1.3.6, https://matplotlib.org/basemap/), with coastlines and political boundaries from GSHHG^44^ and GMT^45^ contributors under the LGPL-3.0 license.

We have tested whether seasonal shifts in SB speed and occurrence reflect climate-driven changes in atmospheric circulation by analyzing trends in anticyclones and cyclones and their correlation with SB. Using an automated gridded Jenkinson–Collison classification (JC), we conducted a regional assessment for each station (see Methods). Our results show a general non-significant decline in anticyclones annually and across most seasons, with the exception of winter, when a widespread and significant increase occurs across the region (Fig. 6a). Conversely, cyclonic circulations are rising across much of the region and timescales, with some exceptions observed in Spain and France during winter and spring (Fig. 6a). These regional patterns are consistent with SB occurrence: anticyclonic conditions favor SB occurrence, whereas cyclonic conditions tend to suppress it (Fig. 6b). The winter increase in anticyclones demonstrates that SB seasonality is extending into winter. For SB speed, our results reveal no significant correlation with anticyclones at the basin scale (Fig. 6c), underscoring the complex role of thermodynamic drivers.

**Fig. 7:**
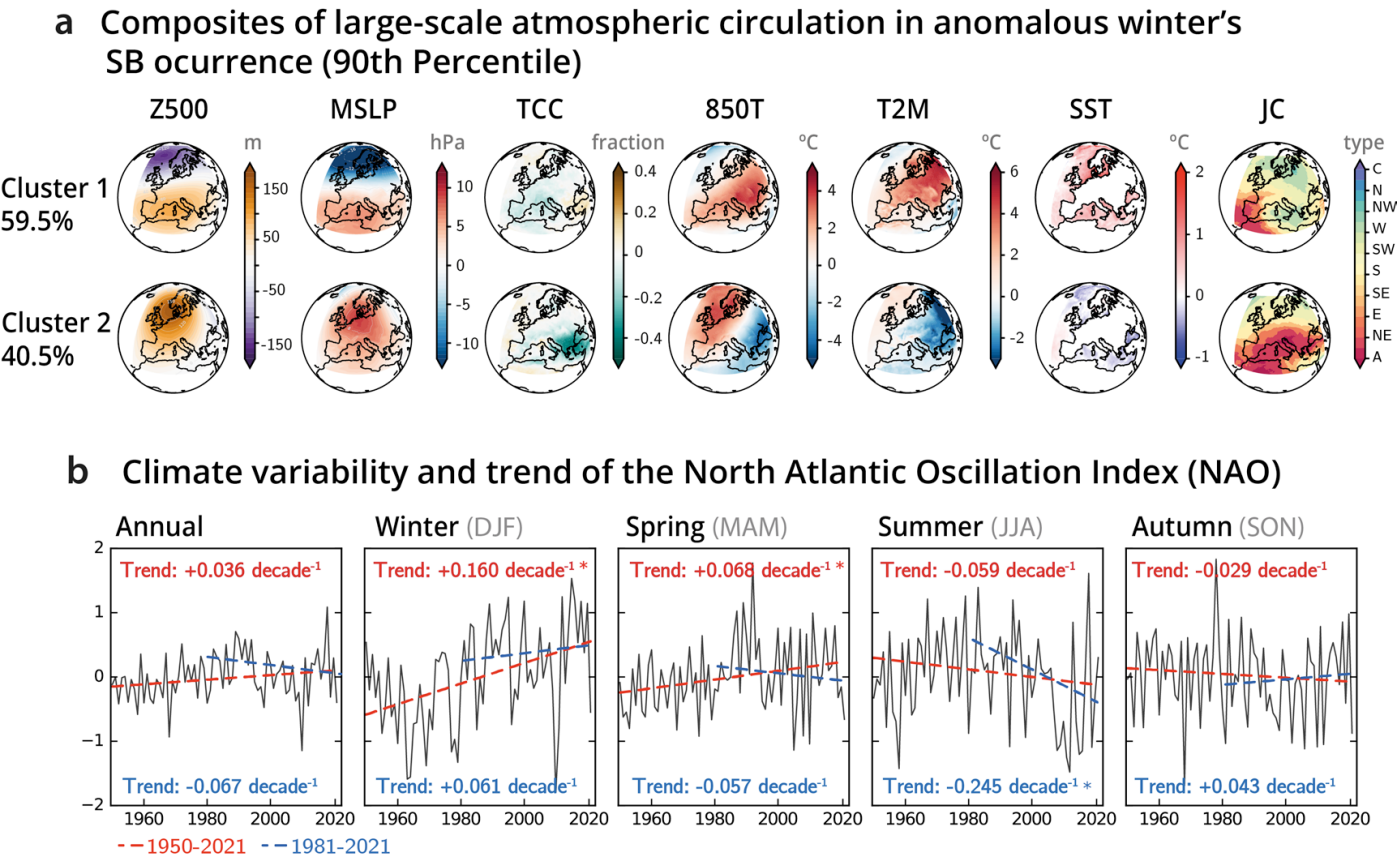
Large-scale circulation patterns associated with anomalous winter SB occurrence. **a** Composites of the two main clusters summarizing ideal conditions for anomalous SB occurrence in winter (90th percentile) in terms of 500 hPa geopotential height (Z500), mean sea-level pressure (MSLP), total cloud cover (TCC), air temperature at 850 hPa (850T), surface air temperature (T2M), sea surface temperature (SST), and Jenkinson–Collison (JC) weather types. **b** Temporal series of the North Atlantic Oscillation Index (NAO) showing the climate variability and trend since 1950. Statistical significance at *p* ≤ 0.05 is reported with an asterisk. Maps in **a** were produced using the Python Matplotlib Basemap Toolkit (v1.3.6, https://matplotlib.org/basemap/), with coastlines and political boundaries from GSHHG^44^ and GMT^45^ contributors under the LGPL-3.0 license.

To better understand the underlying mechanisms, we focus on anomalously high SB occurrence in winter (top 10% of episodes), a season of typically reduced SB frequency (Fig. 7a). We used a clustering approach to summarize the ideal conditions for SB development in winter months, resulting in two main synoptic clusters (C1 and C2) which report SB to primarily develop under high-pressure systems over the Mediterranean—Iberian Peninsula (C1, 59.5% of episodes) and Scandinavian Blocking regimes (C2, 40.5% of episodes). Figure 7a also shows similar synoptic conditions across the two main clusters: positive anomalies in 500 hPa geopotential height (Z500), mean sea-level pressure (MSLP), reduced total cloud cover (TCC), warmer 850T and T2M, anomalously warm Mediterranean SST, and anticyclonic conditions over the Iberian Peninsula (C1) or centered over the Western Mediterranean (C2). C1 conditions are consistent with the Azores High displaced towards the Iberian Peninsula, and positive phases of the North Atlantic Oscillation (NAO+). Figure 7b shows a significant positive winter NAO+ trend since 1950 (also in spring), while since 1981 the trend, although still positive, is no longer significant. This increase aligns with the observed rise in winter SB occurrences across the Mediterranean basin.

## Discussion and summary

Our 41-year observational study suggests that the unprecedented warming of the Western Mediterranean region has likely resulted in weaker SB since 1981, while also pointing to a tendency toward more frequent SB as a response to the interplay between thermodynamic and dynamic mechanisms. This regional-scale assessment highlights the complex nature of understanding SB variability and the link to anthropogenic climate change, and underscores the need for future research to clarify the remaining uncertainties in a warming climate.

We show that the increased radiative forcing^2^, evident in the thermal variables, has significantly amplified the land-sea thermal contrast, which would normally be expected to strengthen SB^46^. Paradoxically, despite a stronger thermal contrast, SB speed have weakened by ~ 10% across the region and time scales, a decline we link to global warming and the intensification of summer heatwaves. The weakening of the SB is particularly critical, as it may reduce their cooling effect and exacerbate heat stress^22^, especially during heatwaves, whose increasing frequency and intensity pose substantial socioeconomic and environmental impacts in the region.

**Fig. 8:**
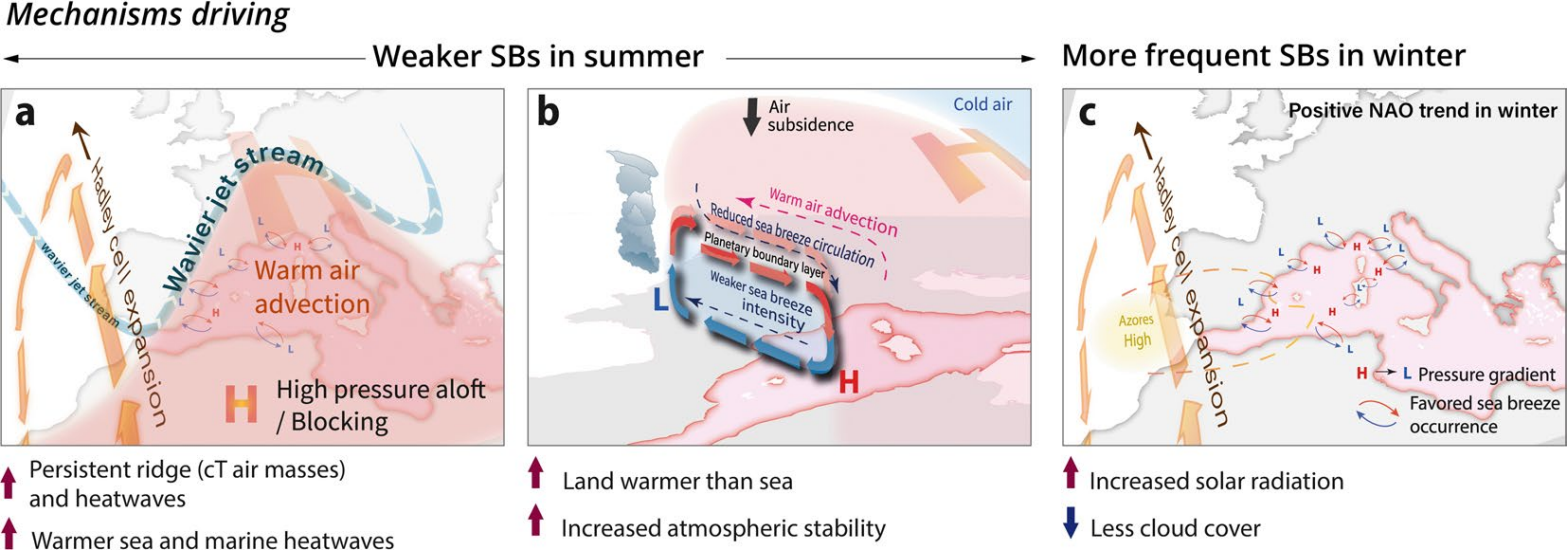
Schematic summary of the thermodynamic and dynamic mechanisms likely contributing to observed SB changes in the Western Mediterranean. **a**, **b** Illustrate how a wavier jet stream transports warm air over the planetary boundary layer, leading to weaker SB in summer. **c** The increased winter SB occurrence is explained by the higher frequency of anticyclones and the positive NAO tendency over the Mediterranean.

We thus hypothesize the following cascading atmospheric events to underlie the weakening of the general SB circulation: with the recent increase in planetary resonance waves, the jet stream is becoming slower and wavier^33^, favoring the development of persistent atmospheric blocking over central Europe and the Mediterranean^47^ (Fig. 8a). This blocking enables the intrusion of warm tropical air masses to mid-latitudes, leading to an increased occurrence of atmospheric heatwaves in the Mediterranean^36^, which is consistent with our findings of higher occurrence and duration of heatwaves. Since atmospheric heatwaves are a key factor behind the reduced summer SB speeds, we associate the weakening to the warm air advection above the planetary boundary layer (PBL), which reinforces subsidence and stabilizes the atmosphere below, resulting in a reduced vertical motion and flattened SB circulation (Fig. 8b). While this hypothesis has been only marginally explored in the literature^34^, our results provide clear evidence of SB circulation responding to these complex thermodynamic and dynamic changes.

As expected, our results also link the increased land-sea thermal contrast to more frequent SB across the basin and throughout almost all seasons. However, it is particularly notable in winter, a season of typically reduced SB activity^25^. Further, with a faster rate of increase compared to spring or summer, our findings suggest that SB occurrence may be undergoing a shift in their seasonal activity, and highlights that thermal forcing alone may not fully explain the recent observed trends. Instead, seasonal changes in atmospheric dynamics may contribute to modulate their occurrence.

We identify a cascading response that further reinforces the evidence that increased winter SB occurrence may be a potential response to regional warming. Our results link the generalized winter increase across the basin to a higher frequency of surface anticyclones over the Western Mediterranean^48,49^. We associate the main pattern displayed in our cluster composites with the influence exerted by the Azores High, which increases the prevalence of winter anticyclones over the Mediterranean^31,48^. The expansion of the Azores High has been linked to a likely widening of the Hadley circulation since the 1980s^2,50,51^ and more frequent NAO+ conditions in winter since at least the 1950s^31^. The underlying connection between SB development, the NAO+, and the anticyclonic stable circulation has already been suggested for the Iberian Peninsula^25^, supporting our interpretation. However, whether the Hadley cell or the NAO phases respond to anthropogenic warming or internal variability is still under debate^51,52^. By promoting a more stratified lower atmosphere through subsidence, anticyclones create conditions of weak synoptic winds, suppressed vertical mixing, and reduced cloud formation. In a warming climate, the observed increase in anticyclones, along with decreasing total cloud cover^53^ and rising solar radiation^54^ are expected across the Mediterranean, being in line with our composite results. Our findings provide evidence that these climate-driven mechanisms may contribute to the observed increase in winter SB occurrence (Fig. 8c).

While the increased winter occurrence of SB is well explained, the mechanisms underlying their weakening during summer heatwaves are more complex and remain less well understood. Besides, the spatial heterogeneity across the basin further complicates the interpretation of regional patterns. For instance, our findings indicate that increased summer cyclone activity may be enhancing easterly advection^35,55^, which suppresses the SB development along the African coast, while simultaneously promoting thermal lows over the Iberian Peninsula^56^ that favor SB triggering. The interaction of SB with orography, larger-scale systems and the sea surface temperature adds further complexity to their behavior.

The radiative effects of aerosols are another mechanism that warrants consideration. Aerosol radiative forcing may potentially contribute to weaken SB, either by altering the land-sea thermal contrast through warming surface temperatures via longwave imbalances^57–59^, or by direct radiative effects that reduce the thermal contrast by attenuating the shortwave absorption and cooling the near-surface^15,23,60^. This latter mechanism reduces the sensible heat flux between the surface and the lower troposphere (e.g., 850 hPa), which diminishes boundary-layer buoyancy and suppresses PBL development^61^, resulting in a weaker SB^60^. These changes are also often associated with lower-tropospheric lapse rate shifts and increased atmospheric subsidence during extreme events, which further stabilize the atmosphere. However, under intense surface heating, enhanced warming in the middle troposphere may increase static stability, acting as a mechanical constraint that limits the influence of surface fluxes on the low-level atmospheric column^62,63^. Given aerosol’s association with altering vertical temperature profiles and atmospheric stability in winter high-pressure systems^56^, or continental air masses in summer^55^, resolving how these non-linear processes interact to shape SB circulation remains a critical challenge for future research.

Weaker but more frequent SB may alter ventilation and pollutant dispersion along densely populated coastal areas. Reduced wind intensity can limit horizontal transport, while more frequent and persistent SB may enhance pollutant recirculation, potentially exacerbating exposure during warm-season episodes. In addition, coastal urbanization and land-use changes can modify surface fluxes, roughness, and moisture availability, potentially interacting with thermodynamic forcing and further influencing SB structure and effectiveness as a ventilation mechanism^22,40,41^. While quantifying these coupled effects requires dedicated investigation, our results highlight the need to consider SB variability within integrated assessments of climate change, urban development, and air-quality management in Mediterranean coastal regions.

Our study provides novel insight by documenting a concurrent weakening of SB speeds and an increase in their occurrence at spatio-temporal scales not previously explored. Regional warming appears to be affecting SB, though the underlying drivers vary across regions; in the Western Mediterranean, the observed changes reflect the interplay between local thermodynamic forcing and synoptic-scale dynamics. This underscores both the importance of local-to-regional observational assessments and the need to extend validation to other SB-sensitive hotspots worldwide. Because global models and reanalyses struggle to capture local wind patterns, and multidecadal assessments are hampered by limited observational coverage, our work represents a major effort to overcome these constraints through four decades of homogenized observations from multiple stations across the Western Mediterranean. Overall, our findings position SB as a critical yet underexplored element of regional climate change, advancing understanding of their evolving patterns in this climate hotspot.

## Methods

### Wind observations

We obtained near-surface wind speed data across the Western Mediterranean basin from the Hadley Integrated Surface Dataset (HadISD version v3.3.1.202301p)^64^ of the Met Office Hadley Centre (available at https://www.metoffice.gov.uk/hadobs/hadisd/; last accessed 01 April 2026). From the original database, we selected 23 meteorological stations (across France, Italy, Tunisia and Algeria) which met the following criteria: (a) located within the first 20 km of the coastline; (b) at least with 41-years of historical records for 1981–2021; (c) hourly records with at least 3 observations during daytime hours (i.e., from 1200 to 2000 UTC); and (d) less than 6 years of missing records in total (i.e., 15% of data). We computed daily averages from (c), while days with missing observations were filled with daily wind speed data (averaged with hourly data from 1200 to 2000 UTC) from the ERA5-Land reanalysis (9 km spatial resolution; available at https://cds.climate.copernicus.eu/; last accessed 01 April 2026)^65^ during the quality control and homogenization procedure.

In addition, we used a sub-daily wind dataset from 16 weather stations located along eastern Spain, originally provided by the Spanish State Meteorological Agency (AEMET) and described in^25^, which we updated to 2021 to cover the full 41-years period. While AEMET is also the main data provider for HadISD in Spain, not all historical records or available measurements are included in HadISD. We therefore opted for the dataset described in^25^, as it originates from the same source, has already been homogenized, validated, and includes five additional weather stations not included in the integrated dataset (Table S1). Although some Spanish stations switched to three-hourly data shortly after the mid-1980s automation^14^, others transitioned later, in the early 2000s. Because of this, we still relied on the sub-daily dataset to ensure consistency across all stations for most of the study period. The dataset derives daily averages from three diurnal observations recorded at synoptic times (i.e., 0700, 1300 and 1800 UTC). It captures the diurnal wind variability without losing the wind signal associated with the SB, since early morning winds preceding a SB day are typically too weak along the Spanish Mediterranean coast^25^. Therefore, the daily dataset is primarily weighted by winds at times when the SB blows (e.g., 1300 and 1800 UTC).

### Homogenization of wind series

To address data inconsistencies and preserve the climate signal, homogenization procedures to the wind speed series were applied following^25^, while a detailed explanation of the process is described in^14^. A total of 39 daily wind speed series were homogenized by applying the CLIMATOL package v.4.1.0 (https://climatol.eu/; last accessed 01 April 2026)^66^, a tool widely used in wind research for quality control, detect inhomogeneities and missing data infilling of climate records. The homogenization is based on 10-meters reference wind speed from both observations and ERA5-Land, prioritizing neighbor stations when available. The procedure did not detect any outlier but corrected 65 break points in overall with SNHT > 25. The resulting dataset contains 39 weather stations (Fig. S1 and Table S1) with daily wind speed series spanning the 1981–2021 period.

### Identification of days with SB events

Traditional methods identifying SB relies on well-defined criteria on local variables at high temporal resolution, but observational databases lack spatial-temporal hourly data to address long-term studies. For this reason, we designed an automated algorithm capable of identifying potential SB events based on (a) synoptic and (b) local weather conditions from reanalysis data. Our method allows the detection at any time and location of the Western Mediterranean basin by taking into account 5 objective criteria that remove days driven by prominent synoptic disturbances (filters 1–2) and assure the presence of pure SB events taking place locally (filters 3–5).

- **Filter (1)** Discards days dominated by cyclonic weather types (CW, CNW, CE, CNE, CN, CS, CSE, CSW) from the Jenkinson and Collison’s weather scheme (hereafter JC)^67^. Pure C conditions were maintained since summer thermal lows enhance SB, particularly over the Iberian Peninsula and North Africa^15^. To obtain the JC, we followed^48^ by applying the Python’s module “jcclass” (https://github.com/PedroLormendez/jcclass; last accessed 01 April 2026)^68^ to sea level pressure (MSLP) gridded data from the ERA5^69^. We reclassified U type as A (MSLP > 1020 hPa) and C (MSLP < 1020 hPa) according to^70^. The reduced 10 weather types scheme is based on the dominant advection: A, NE, E, SE, S, SW, W, NW, N, and C.
- **Filter (2)** Select days with weak geostrophic winds (≤ 12 m s^−1^)^25^. They were computed from 850 hPa geopotential fields at 1200 UTC from the ERA5 reanalysis^69^.
- **Filter (3)** Consider winds blowing from the sea based on the coastal orientation of each station and the wind direction between 1200 and 1700 UTC (at least one hour) from ERA5-Land. Additionally, to account sideshore SB, we expanded the orientations listed in Table S1 by ± 15°. For the Balearic and Eastern islands, we consider all wind directions (i.e., 0º–360º) following^25^.
- **Filter (4)** Choose days with a dominance of clear skies by setting the cloud coverage < 4 oktas (i.e., 56.25% cloudiness)^71^ at 1200 UTC from ERA5^69^.
- **Filter (5)** Filter out rainy days (daily precipitation < 0.1 mm day^−1^)^72^ from ERA5-Land, as SB days are not associated with precipitation (except for the development of fronts which mainly bring rain inland).

We justify using ERA5 in Filters 2 and 4 by the availability of data at vertical levels (upper air), since ERA5-Land is primarily focused on near-surface variables. The final SB database for the Western Mediterranean basin contains 41-years of SB events for each station shown in Fig. S1. In this work, we identified a total of 3,544 days regionally (around 23% of the days between 1981 and 2021), while the number of days identified by coast and weather stations is listed in Table S2.

### Identification of atmospheric heatwaves

We identified atmospheric heatwaves following the CTX90 approach^73^, which defines a heatwave as a period of at least three consecutive days where daily maximum air temperatures exceed the 90th percentile, calculated within a 15-day moving window for each calendar day. We used daily maximum and minimum air temperature data from the ERA5-Land reanalysis to detect heatwaves at station level during the summer season (June-August) from 1981 to 2021. After identifying historical heatwaves over the Mediterranean region, we created a heatwave dataset based on the following metrics: (a) annual number of heatwave events (HWN), (b) heatwave frequency or total annual heatwave days (HWF), (c) maximum heatwave duration (HWD) - based on the annual longest event, and (d) average heatwave duration (HWDM). We also generated an observational heatwave database based on daily air temperature observations from the HadISD dataset. This second dataset, which includes metrics for 34 weather stations, was developed to assess whether the heatwave patterns derived from reanalysis are consistent with those from observations. The two datasets show strong agreement, despite minor discrepancies at a few stations (Fig. S8).

### Data analysis

For all analyses, we first derived monthly averages from daily means of SB days at station level. Anomalies were then computed by subtracting the 1981–2021 monthly climatology from the monthly averages. Subsequently, they were resampled at two timescales: annually and boreal seasons (winter – DJF, spring – MAM, summer – JJA, autumn – SON). Further, the analyses were conducted at three spatial scales: at regional level, by averaging all SB days identified for all stations across the Western Mediterranean basin; at coastal level, by grouping stations with common variability (Fig. S1); and at weather station level, as described in Table S1.

### Trends

We estimated decadal trends for SB speeds (in m s^−1^ decade^−1^) and occurrence (in days decade^−1^) by applying a linear regression to the time series. It is widely known that trend analyses in terms of magnitude and significance are sensitive to the period length. For this reason, and to understand better the persistence of the changes along the 41-year assessment, we conducted running trend analyses at regional level, by estimating trends in moving windows from 10-years onwards. We also estimated gridded trends of warming variables by applying a linear regression on each pixel along the time dimension. The warming variables were defined as: surface air temperature (T2M), air temperature at 850 hPa (850T), sea surface temperature (SST), solar net radiation (SR) and the land-sea temperature contrast (∆T) from the ERA5 reanalysis^69^. ∆T was calculated as the difference between the T2M over a representative land point and a representative point over the sea for each location. Trends on ∆T, heatwaves (number and duration), and NAO Index were also assessed at each weather station level. Overall, changes are expressed as changes per decade (i.e., decade^−1^), but also as relative percent of change per decade. We assessed statistical significance at two *p*-level thresholds: significant at *p* ≤ 0.05, and non-significant at *p* > 0.05.

### Relationship between warming, JC and the SB

To explore the relationships between the warming variables and the SB speed and occurrence, we performed Pearson correlation analysis. We used the Pearson correlation coefficients to quantify the strength and direction of linear associations between variables and the detrended anomalies of the SB. In addition, to better understand the impact of a warmer Mediterranean on the SB, we grouped their speed and occurrence according to four warming levels (< 0.5 °C, 0–1 °C, 1–1.5 °C, and > 1.5 °C) based on surface temperature anomalies (T2M, relative to the 1951–1980 baseline) from ERA5 reanalysis, for 1981–2021. We applied ANOVA and Tukey’s HSD post-hoc tests to the annual and seasonal means to assess the significance of the differences among warming levels.

### Effects of heatwaves on the SB speeds

We assessed the mean differences in SB speeds between heatwave (HW) and non-heatwave (NHW) days. For each station and coast, we performed a *t-test* on daily SB observations (excluding missing values). To ensure statistical significance and account for multiple comparisons across stations, we adjusted the *p*-values using the Holm correction.

### Large-scale atmospheric circulation patterns

We characterized the large-scale circulation on the most anomalous winter months of SB occurrence (90th percentile) by employing a clustering analysis. This analysis utilized Z500, MSLP, TCC, T2M, 850T, SST, and JC data from ERA5^69^, which were standardized before performing k-means clustering (k = 2) to identify dominant circulation patterns. Cluster composites were then constructed by averaging anomalies within each cluster.

## Supplementary Information

Below is the link to the electronic supplementary material.

Supplementary Material 1.

## Data Availability

The near-surface wind speed used to quantify SB speed changes were obtained from the HadISD [v3.3.1.202301p, available at (https://www.metoffice.gov.uk/hadobs/hadisd/)] on an hourly basis, complemented with additional records from Spanish stations provided upon request by AEMET. The complete list of weather stations is provided in Table S1 of the supplementary information. The detection of SB, as well as the analyses of thermal variables and large-scale circulation, were carried out using the ERA5 and ERA5-Land datasets [available at (https://cds.climate.copernicus.eu/)]. The heatwave database was also constructed using HadISD and ERA5-Land. The processed 41-year observational SB database (on a daily basis) along with monthly aggregates and related-datasets to reproduce our results are publicly available at (10.6084/m9.figshare.30582344).

## Code availability

All analysis were generated with the open-source software Python v3.8.15 and R. For the homogenization procedure we used the CLIMATOL package (https://climatol.eu/*).* In addition, we performed statistical analysis using the following Python packages: (1) xarrayutils to quantify gridded linear regressions of thermal variables (https://xarrayutils.readthedocs.io/en/latest/utils.html), (2) hotspell to identify atmospheric heatwaves (https://github.com/agathangelidis/hotspell), (3) jcclass^68^ to derive the gridded JC classification (https://github.com/PedroLormendez/jcclass), (4), scipy to compute the linear regressions for the trend assessment (https://docs.scipy.org/doc/scipy/reference/stats.html), (5) pingouin to perfom the statistical pairwise t-test (https://pingouin-stats.org/build/html/index.html), and (6) statsmodels for the Tukey’s HSD post-hoc test (https://www.statsmodels.org/stable/index.html). The source code to reproduce the main figures is publicly accessible at: https://doi.org/10.6084/m9.figshare.30582344.
