# Supplementary material for: Weaker and more frequent Mediterranean sea breezes in a warming climate
Source: Sci Rep. 2026 May 15;16:25266. doi: 10.1038/s41598-026-47025-4 (PMC13470010; doi:10.1038/s41598-026-47025-4)
Supplement: Supplementary file 1 [file 41598_2026_47025_MOESM1_ESM.pdf]

Weaker and more frequent Mediterranean sea breezes in a warming climate

Shalenys Bedoya-Valestt[1*], Cesar Azorin-Molina[1], Nuria P. Plaza-Martin[1], Lorenzo Minola[2], Luis Gimeno[3,4], Miguel Andres-Martin[1], Sergio M. Vicente-Serrano[5], and Deliang Chen[6]

[1] Centro de Investigaciones sobre Desertificación, Consejo Superior de Investigaciones Científicas (CIDE, CSIC-UV-GVA), Climate, Atmosphere and Ocean Laboratory (Climatoc-Lab), Moncada, Valencia, Spain

[2] Interuniversity Department of Regional and Urban Studies and Planning (DIST), Politecnico and University of Turin, Turin, Italy

[3]Centro de Investigación Mariña, Environmental Physics Laboratory (EPhysLab), Universidade de Vigo, Ourense, Spain

[4]Galicia Supercomputing Center (CESGA), Santiago de Compostela, Spain

[5]Instituto Pirenaico de Ecología, Consejo Superior de Investigaciones Científicas (IPE-CSIC), Zaragoza, Spain

[6]Ministry of Education Key Laboratory for Earth System Modeling, Department of Earth System Science, Tsinghua University, Beijing 100084, China

*Corresponding author: Shalenys Bedoya-Valestt. E-mail: shalenys.bedoya@csic.es. ORCID-ID: 0000-0001-7386-9127. Address: Centro de Investigaciones Sobre Desertificación, Consejo Superior de Investigaciones Científicas (CIDE, CSIC-UV-GVA), Ctra. CV-315 Km 10.7, 46113 Moncada, Valencia, Spain.

List of supplementary figures

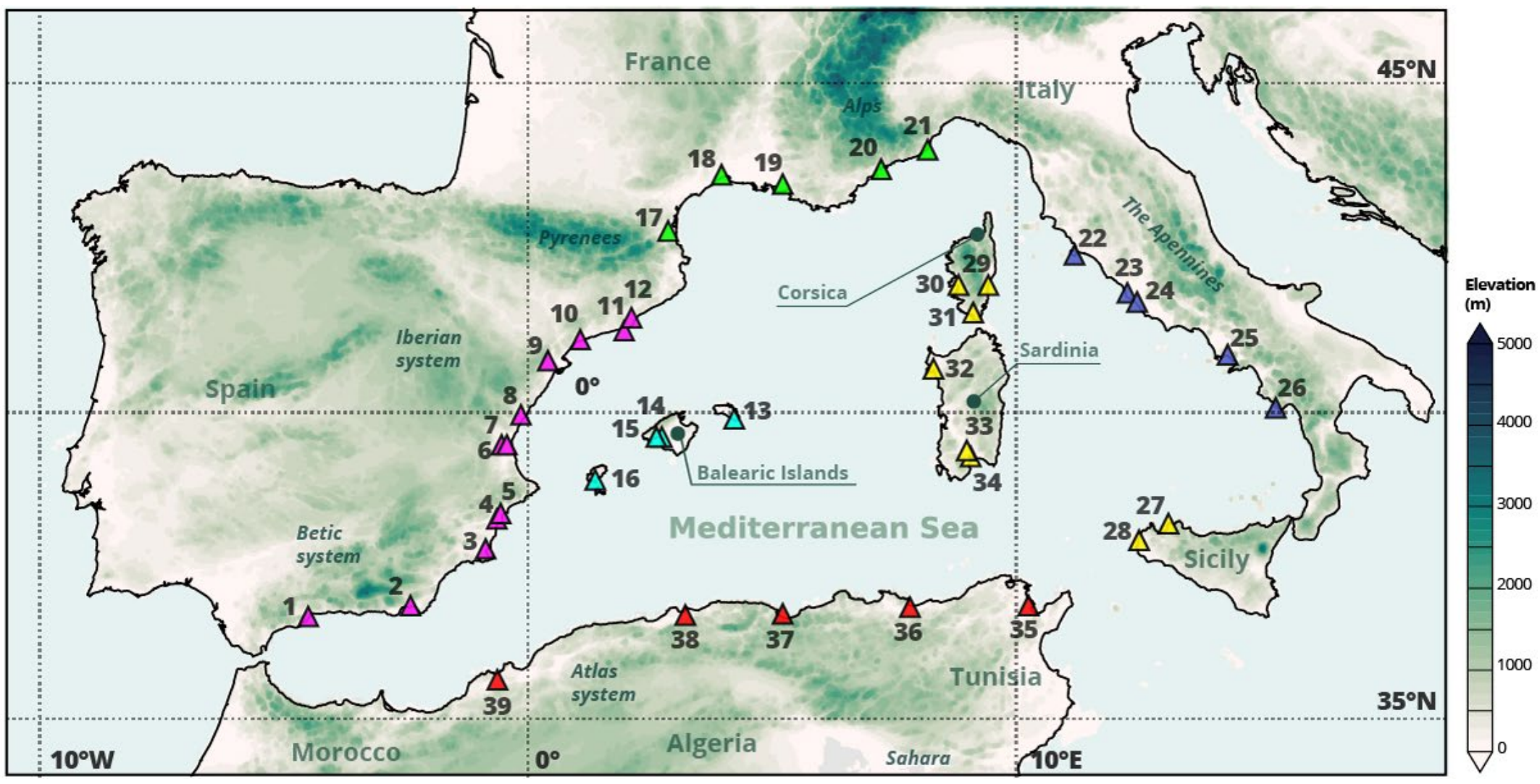

Fig. S1: Terrain map of the Western Mediterranean basin with location of the 39 weather stations (triangles); the numbers are described in Table S1. Spanning a coastline of 4,673 km along five countries (Spain, France, Italy, Tunisia and Algeria), stations are grouped by coastal regions: Spain (magenta), Balearic Islands (cyan; Ibiza, Mallorca and Menorca), France (green), Italy (blue), Eastern Islands (yellow; Corsica, Sardinia and Sicily), and North Africa (red). Maps were produced using the Python Matplotlib Basemap Toolkit (v1.3.8, https://matplotlib.org/basemap/). Coastlines and political boundaries are from GSHHG and GMT contributors under the LGPL-3.0 license. Terrain base maps incorporate SRTM15+ V2.4 land data and were derived from the GEBCO Compilation Group (2022) GEBCO_2022 Grid (https://doi.org/10.5285/e0f0bb80-ab44-2739-e053-6c86abc0289c).

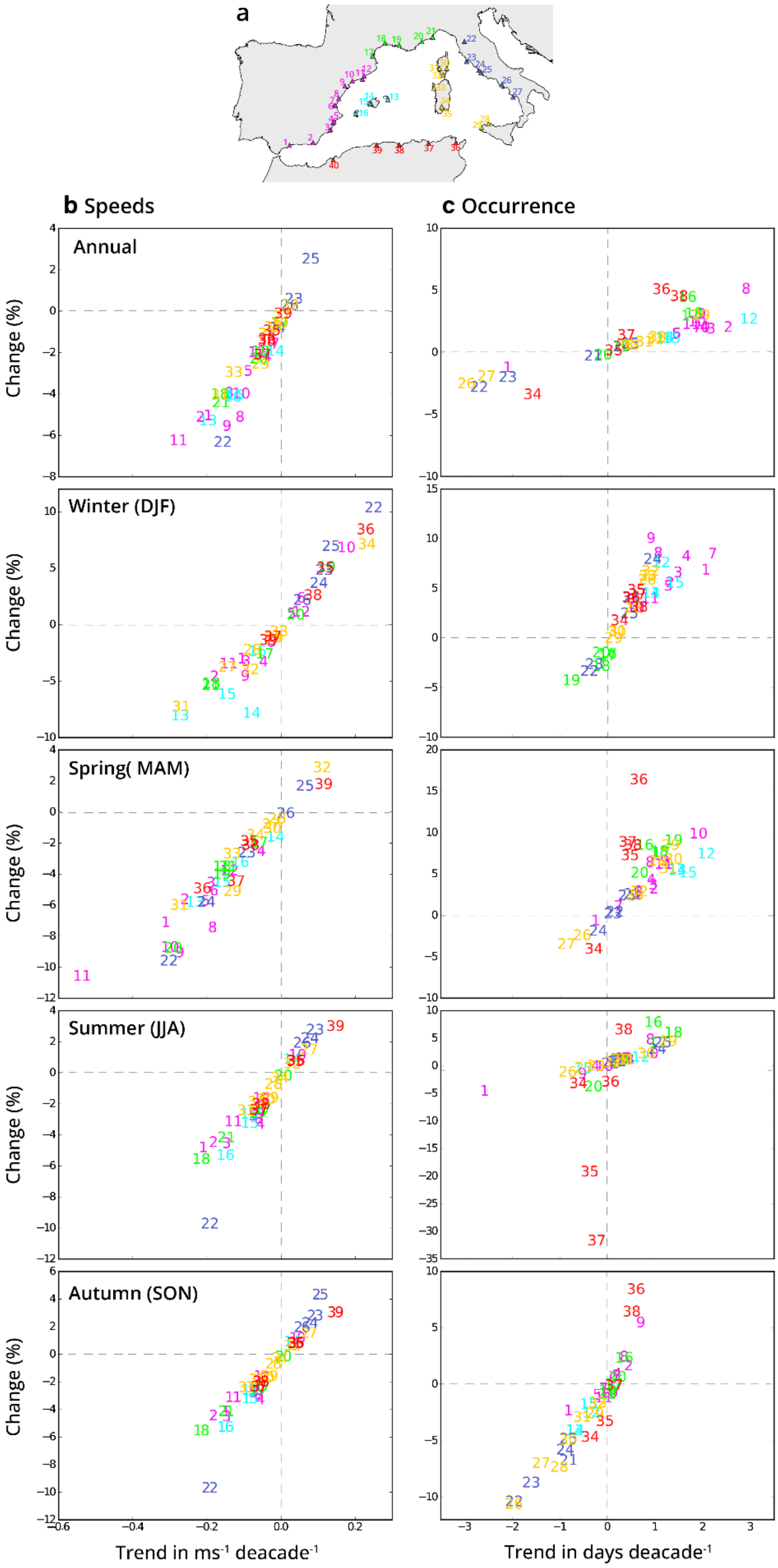

Fig. S2: Percentage of change vs trend by **(a)** weather station for the SB **(b)** speed and **(c)** occurrence for 1981-2021.

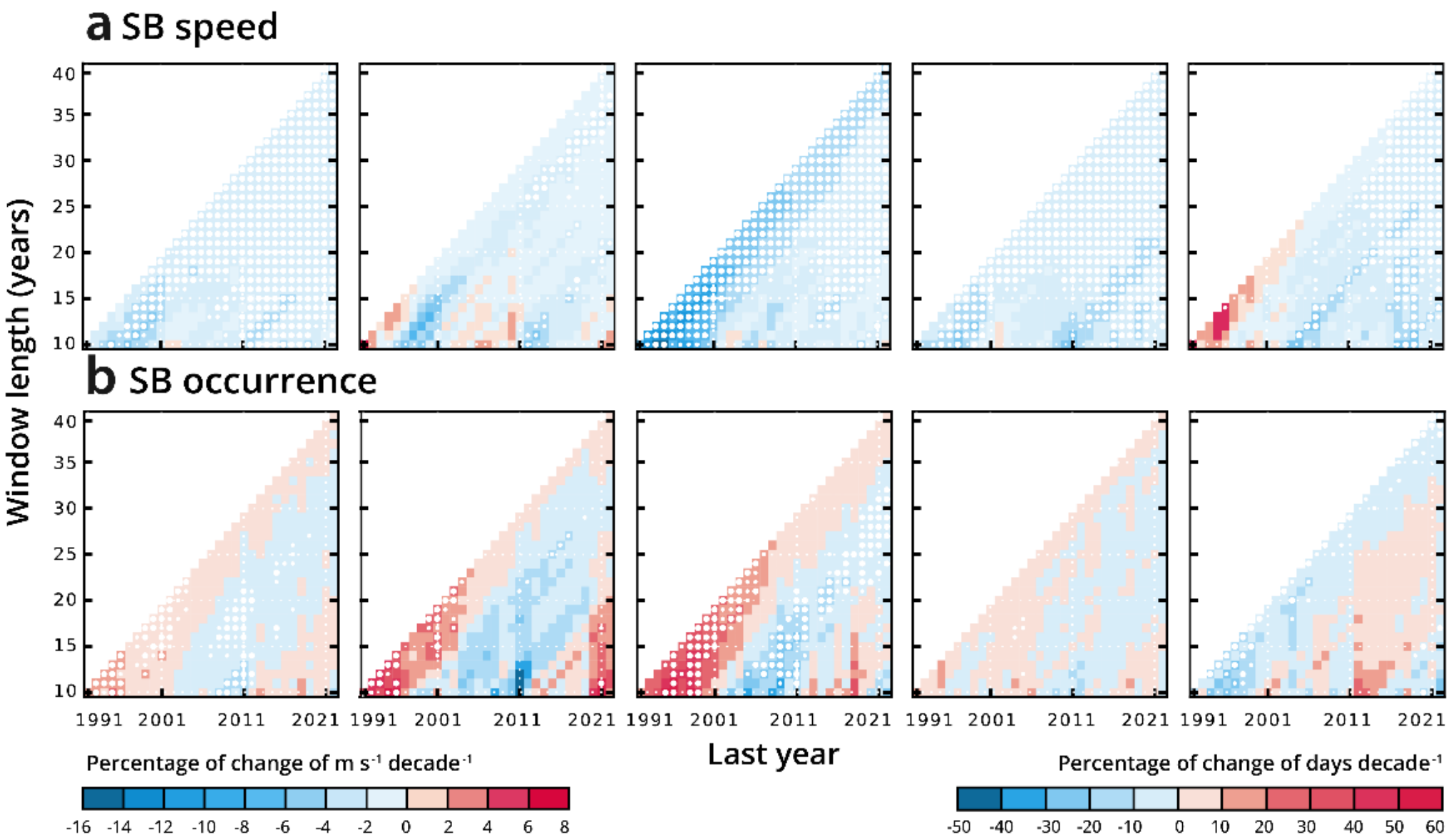

Fig. S3: Regional percentage of change per decade and trend persistence of SB **(a)** speed (m s^{-1}) and **(b)** occurrence (days) for different multiyear-sized running windows. White dots represent significant trends at $p \leq 0.05$.

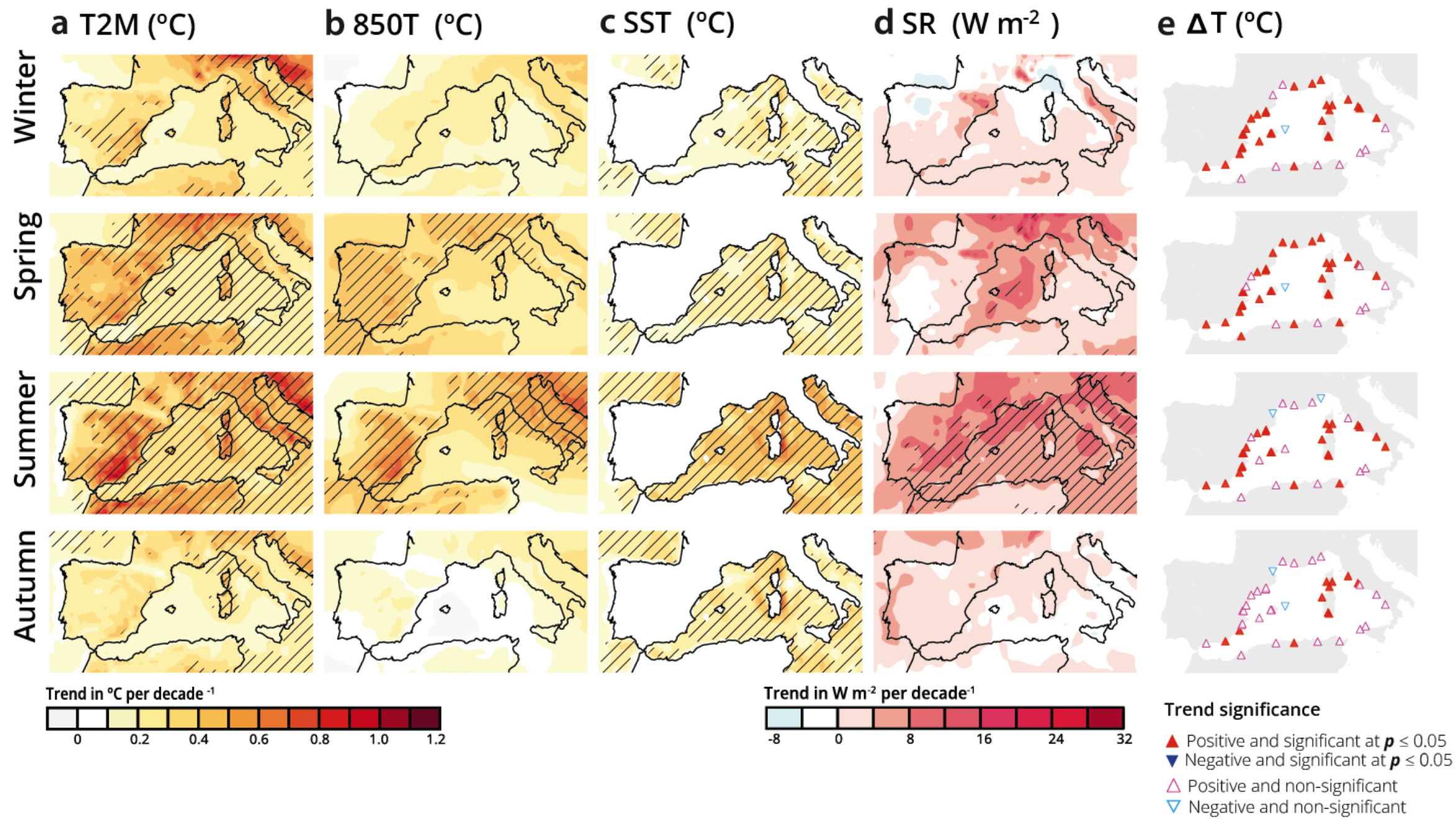

Fig. S4: Changes in warming-related variables for 1981-2021. Spatial seasonal trends of **(a)** surface air temperature (T2M), **(b)** air temperature at 850 hPa (850T), **(c)** sea surface temperature (SST), **(d)** solar net radiation (SR), and **(e)** the land-sea temperature contrast (ΔT). Hatches indicate areas with statistically significant trends at $p \leq 0.05$. Maps were produced using the Python Matplotlib Basemap Toolkit (v1.3.8, https://matplotlib.org/basemap/). Coastlines and political boundaries are from GSHHG and GMT contributors under the LGPL-3.0 license.

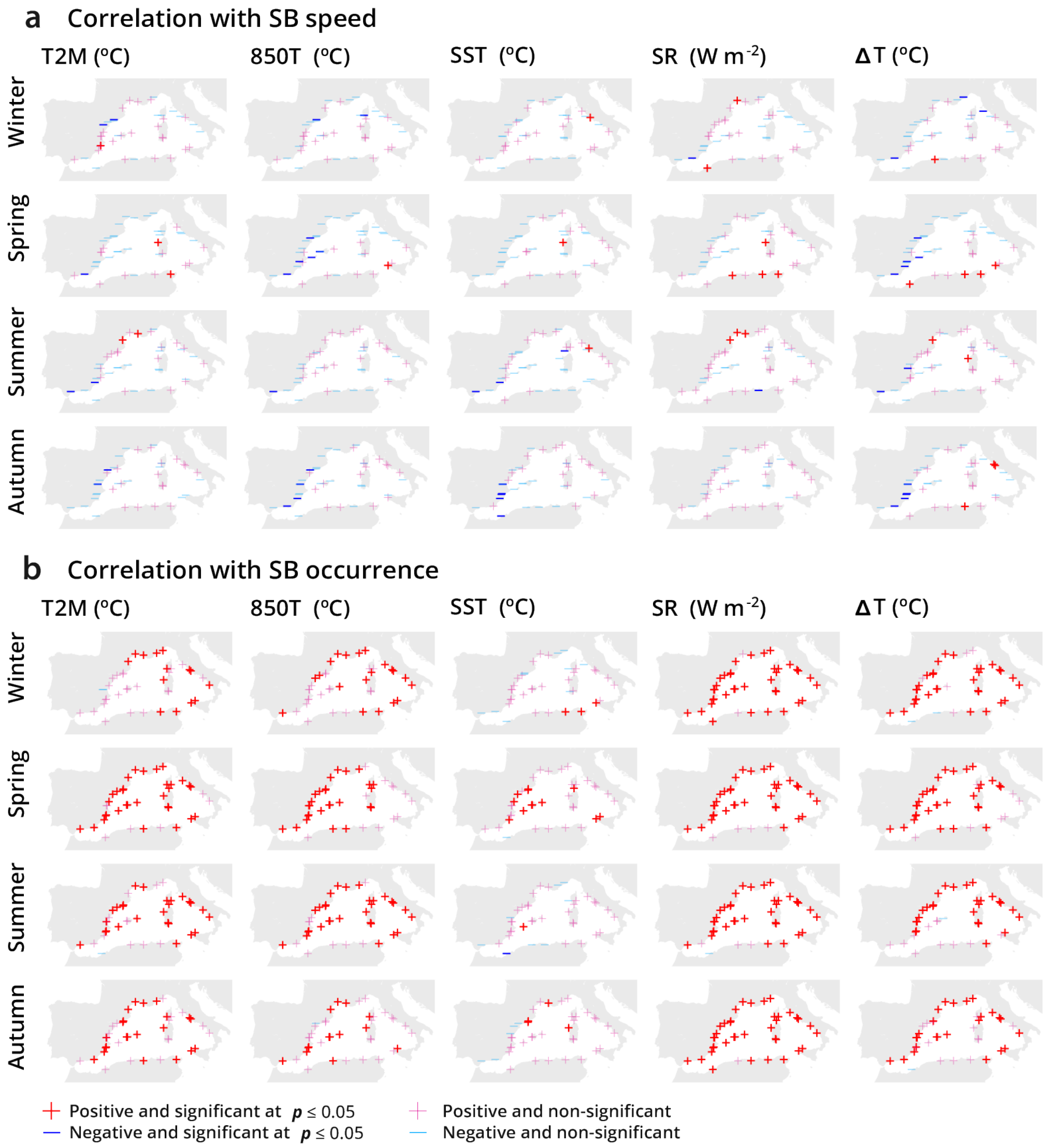

Fig. S5: Correlation of SB with warming-related variables across the Western Mediterranean Basin (1981–2021). Spatial and seasonal distribution of the Pearson's correlations between detrended anomalies of SB **(a)** speed and **(b)** occurrence with surface air temperature (T2M), air temperature at 850 hPa (850T), sea surface temperature (SST), solar net radiation (SR) and the land-sea temperature contrast (ΔT). Statistically significant correlations are highlighted ($p \leq 0.05$). Maps were produced using the Python Matplotlib Basemap Toolkit (v1.3.8, https://matplotlib.org/basemap/). Coastlines and political boundaries are from GSHHG and GMT contributors under the LGPL-3.0 license.

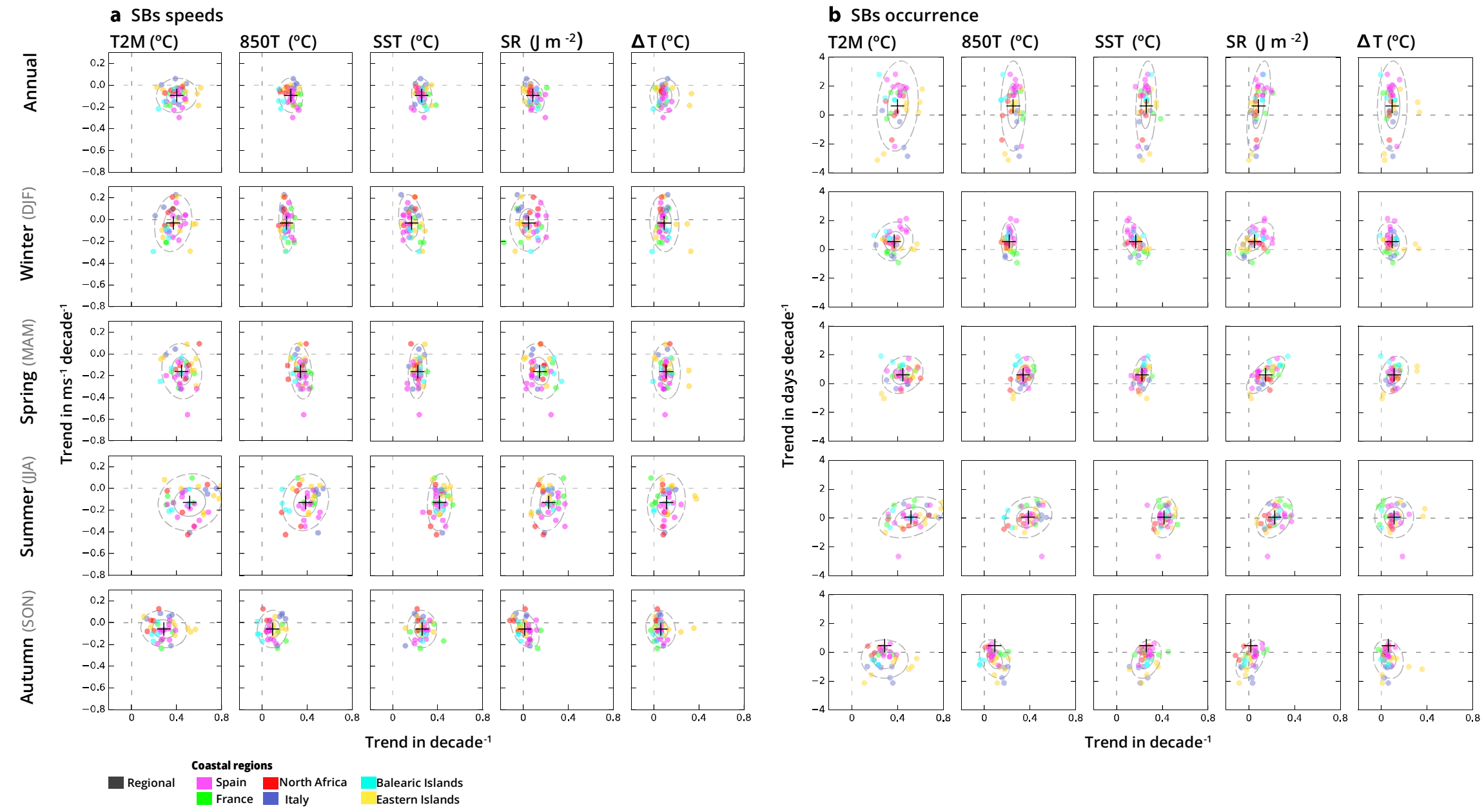

Fig. S6: Trends of warming variables vs. SB trends for 1981–2021. Scatter plots of annual and seasonal trends of warming variables (x axes): surface air temperature (T2M), air temperature at 850 hPa (850T), sea surface temperature (SST), solar radiation (SR), and thermal contrast (ΔT), *versus* the SB (y axes): **(a)** speed (in m s^{-1} $decade^{-1}$) and **(b)** occurrence (in days $decade^{-1}$) for regional and station data points for 1981-2021. Trends are expressed in ºC $decade^{-1}$ for temperature and J m^2 $decade^{-1}$ for solar radiation. Ellipses show confidence regions at 2 standard deviations around the mean: 1σ (68%, solid line) and 2σ (95%, dashed line).

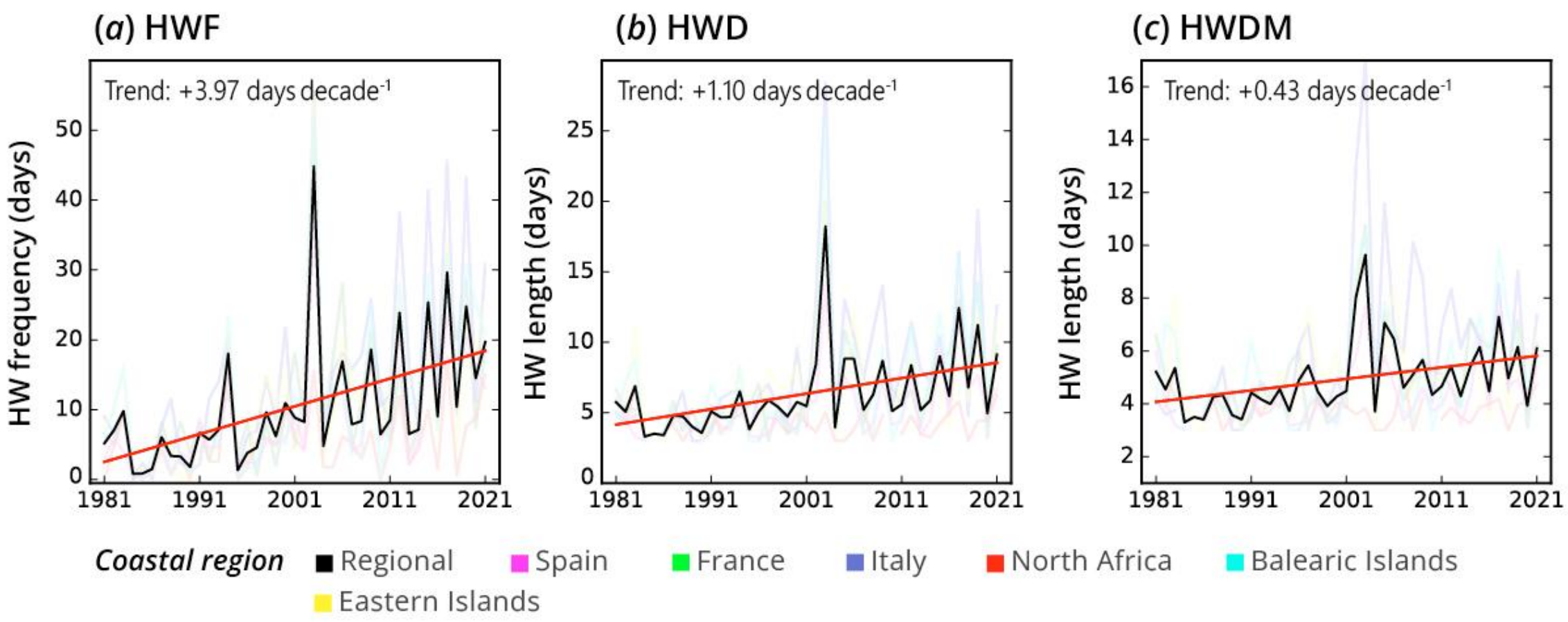

Fig. S7: Trends in heatwaves frequency of occurrence (in days, HWF), heatwaves maximum duration (HWD) and heatwaves mean duration (HWDM).

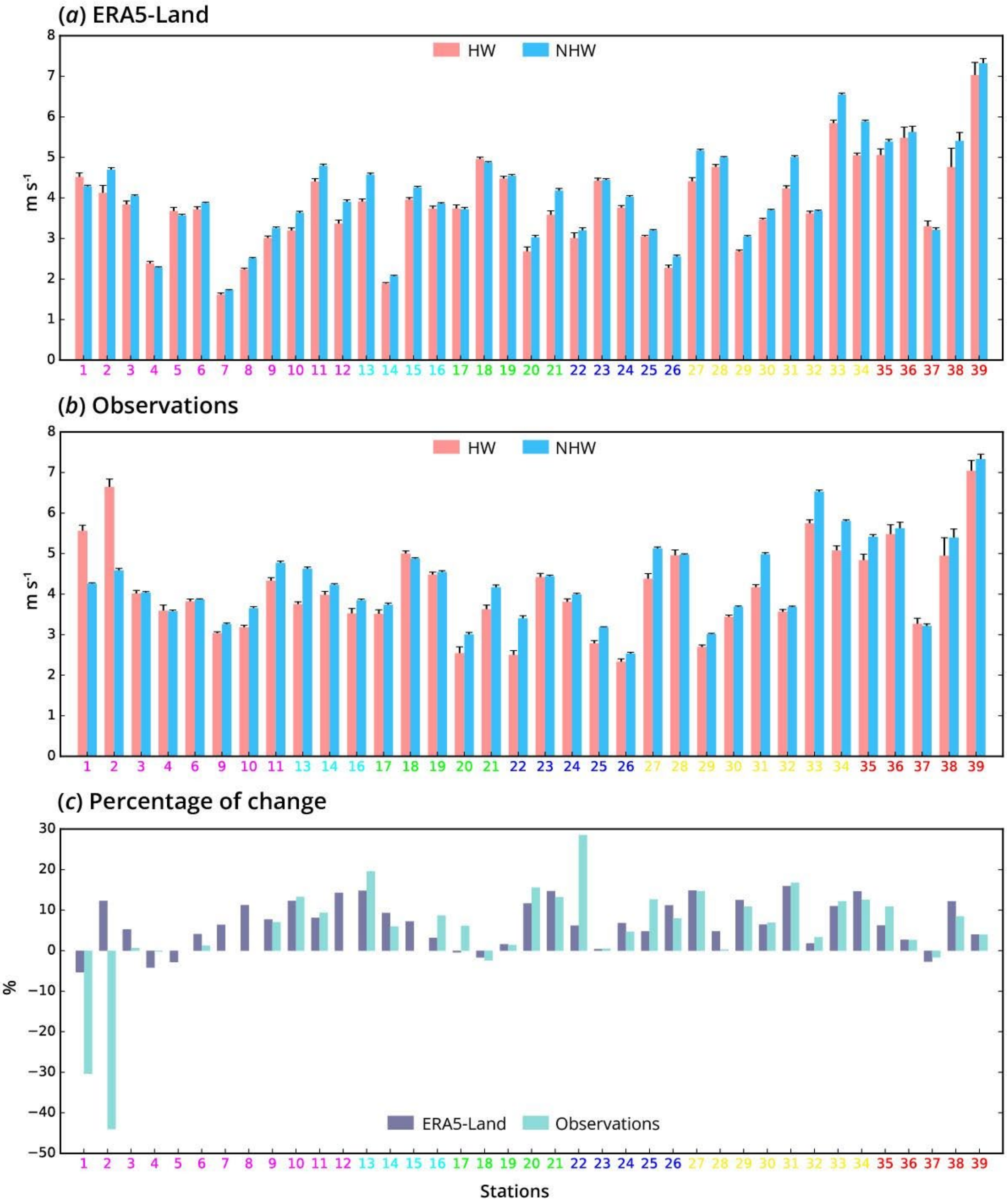

Fig. S8: SB speed during heatwaves (HW) and non-heatwaves episodes (NHW) by weather station. (a) SB speed during heatwaves episodes obtained from ERA5-Land. **(b)** SB speed during heatwaves episodes obtained from observations from the HadISD dataset. **(c)** Percentage of change of the SB speed during HW minus NHW episodes.

List of supplementary tables

Table S1. Description of the meteorological stations with homogeneous wind speed measurements over the Western Mediterranean basin (for locations see Fig. 1) for 1981–2021. Relocated stations in asterisk. Stations are grouped by coastal regions in colours as follows: Spain (magenta), Balearic Islands (cyan; Ibiza, Mallorca and Menorca), France (green), Italy (blue), Eastern Islands (yellow; Corsica, Sardinia and Sicily), and North Africa (red).

Nº	Id	Station	Lat. (Decimal º)	Long. (Decimal º)	Elevat. a.s.l. (m.)	Dist. Coast (km)	Orient. (Decimal º)	Period of records
1	6155A	Málaga airport	36.67	-4.48	5	1.9	75 - 255	1961 - 2021
2	6325O	Almería airport	36.85	-2.36	21	1	75 - 255	1968 - 2021
3	7031	San Javier airport	37.79	-0.80	4	4.5	43 - 223	1961 - 2021
4	8019	Alicante airport	38.28	-0.57	43	4.4	43 - 223	1961 - 2021
5	8025	Alicante Ciudad Jardín	38.37	-0.49	81	2.8	43 - 223	1961 - 2021
6	8414A	Valencia airport	39.48	-0.47	56	12.7	13 - 193	1961 - 2021
7	*8416Y	Valencia Viveros	39.48	-0.36	11	3.2	13 - 193	1961 - 2021
8	*8500A	Castellón-Almassora	39.96	-0.07	43	6.7	13 - 193	1976 - 2021
9	9981A	Tortosa	40.82	0.49	50	18	13 - 193	1961 - 2021
10	0016A	Reus airport	41.15	1.16	71	7.5	49 - 229	1961 - 2021
11	0076	Barcelona airport	41.29	2.07	4	1.5	49 - 229	1961 - 2021
12	0200E	Barcelona Fabra	41.42	2.12	408	8	49 - 229	1961 - 2021
13	B893	Menorca airport	39.85	4.22	91	3	0 -360	1965 - 2021
14	B278	Palma Mallorca airport	39.56	2.74	8	3.7	0 - 360	1972 - 2021
15	B228	Palma Mallorca port	39.56	2.63	3	0	0 - 360	1978 - 2021
16	B954	Ibiza airport	38.88	1.38	6	2.8	0 - 360	1961 - 2021
17	077470	Rivesaltes	42.74	2.87	43.9	14	8 - 188	1973 - 2021
18	076430	Mediterranee	43.58	3.96	5.2	3.8	70 - 250	1973 - 2021
19	076500	Provence	43.44	5.21	22.6	10.6	70 - 250	1973 - 2021
20	076900	Côte d'Azur	43.66	7.22	3.7	0.5	53 - 233	1973 - 2021
21	161530	Cape Mele	43.95	8.17	221	0	53 - 233	1973 - 2021
22	161680	Mount Argentario	42.38	11.17	632	1.7	133 - 313	1973 - 2021
23	162420	Fiumicino	41.80	12.25	4.6	2.8	133 - 313	1973 - 2021
24	162450	Practica di Mare	41.66	12.45	12.5	1.8	133 - 313	1973 - 2021
25	*162890	Napoli/Capodichino	40.85	14.30	72	1.4	133 - 313	1973 - 2021
26	163100	Cape Palinuro	40.02	15.28	185	0	133 - 313	1973 - 2021
27	164050	Palermo	38.18	13.09	19.8	0.5	0 - 360	1973 - 2021
28	164290	Trapani Birgi	37.91	12.29	7.3	1.8	0 - 360	1973 - 2021
29	077650	Solenzara	41.92	9.41	8.5	0.7	0 - 360	1983 - 2021
30	077610	Campo dell Oro	41.92	8.80	5.5	1.3	0 - 360	1973 - 2021
31	077800	Sud Corse	41.50	9.10	26.5	6.2	0 - 360	1973 - 2021
32	165200	Alguero	40.63	8.29	26.5	3.6	0 - 360	1973 - 2021
33	165460	Decimomannu	39.35	8.97	30.5	19	0 - 360	1973 - 2021
34	165600	Elmas	39.25	9.05	4	5.7	0 - 360	1973 - 2021
35	607150	Carthage	36.85	10.23	6.7	11	152 - 332	1973 - 2021
36	603600	Annaba	36.82	7.81	4.9	3.2	87 - 267	1973 - 2021
37	604020	Soummam	36.71	5.21	6.1	0	87 - 267	1973 - 2021
38	603900	Houari Boumediene	36.69	3.22	25	7.1	87 - 267	1961 - 2021
39	604900	Es Senia	35.62	-0.62	89.9	9.8	72 - 252	1961 - 2021

Table S2. Number of days that were discarded (non-sea breeze, NSB) or passed (SB) the filters 1-5 by each weather station, regionally and for coastal regions during the 1981-2021 period.

Nº	Id	Station	Filter 1		Filter 2		Filter 3		Filter 4		Filter 5	
			NSB	SB	NSB	SB	NSB	SB	NSB	SB	NSB	SB
1	6155A	Málaga airport	812	14,163	2,495	11,668	737	10,931	3,325	7,606	2,898	**4,708**
2	6325O	Almería airport	1,030	13,945	2,607	11,338	133	11,205	3,196	8,009	2,062	**5,947**
3	7031	San Javier airport	856	14,119	2,453	11,666	279	11,387	3,217	8,170	2,409	**5,761**
4	8019	Alicante airport	645	14,330	3,163	11,167	448	10,719	3,479	7,240	2,734	**4,506**
5	8025	Alicante Ciudad Jardín	668	14,307	2,196	12,111	785	11,326	4,053	7,273	1,965	**5,308**
6	8414A	Valencia airport	724	14,251	3,121	11,130	899	10,231	3,603	6,628	1,987	**4,641**
7	8416Y	Valencia Viveros	724	14,251	3,121	11,130	680	10,450	3,675	6,775	2,198	**4,577**
8	8500A	Castellón-Almassora	750	14,225	2,230	11,995	385	11,610	4,515	7,095	2,784	**4,311**
9	9981A	Tortosa	686	14,289	3,984	10,305	2,051	8,254	3,272	4,982	2,580	**2,402**
10	0016A	Reus airport	710	14,265	2,537	11,728	2,334	9,394	3,720	5,674	2,897	**2,777**
11	76	Barcelona airport	740	14,235	2,137	12,098	735	11,363	4,864	6,499	3,284	**3,215**
12	0200E	Barcelona Fabra	740	14,235	2,137	12,098	735	11,363	4,845	6,518	3,191	**3,327**
13	B893	Menorca airport	591	14,384	3,386	10,998	0	10,998	3,302	7,696	2,893	**4,803**
14	B278	Palma Mallorca airport	594	14,381	3,001	11,380	0	11,380	4,087	7,293	2,612	**4,681**
15	B228	Palma Mallorca port	594	14,381	3,001	11,380	0	11,380	4,087	7,293	2,612	**4,681**
16	B954	Ibiza airport	864	14,111	2,705	11,406	0	11,406	3,418	7,988	2,172	**5,816**
17	77470	Rivesaltes	784	14,191	6,382	7,809	2,415	5,394	2,641	2,753	1,256	**1,497**
18	76430	Mediterranee	1,015	13,960	4,739	9,221	2,757	6,464	2,939	3,525	1,103	**2,422**
19	76500	Provence	1,008	13,967	4,748	9,219	3,340	5,879	2,528	3,351	978	**2,373**
20	76900	Côte d'Azur	873	14,102	2,465	11,637	133	11,504	4,933	6,571	3,821	**2,750**
21	161530	Cape Mele	899	14,076	2,944	11,132	1,188	9,944	4,363	5,581	3,424	**2,157**
22	161680	Mount Argentario	1,510	13,465	2,697	10,768	1,188	9,580	3,053	6,527	3,266	**3,261**
23	162420	Fiumicino	1,415	13,560	2,523	11,037	833	10,204	3,516	6,688	2,899	**3,789**
24	162450	Practica di Mare	1,373	13,602	2,515	11,087	697	10,390	3,354	7,036	2,997	**4,039**
25	162890	Napoli/Capodichino	1,255	13,720	2,649	11,071	1,635	9,436	3,389	6,047	2,759	**3,288**
26	163100	Cape Palinuro	1,104	13,871	2,841	11,030	679	10,351	3,855	6,496	3,447	**3,049**
27	164050	Palermo	1,075	13,900	2,679	11,221	0	11,221	3,418	7,803	3,252	**4,551**
28	164290	Trapani Birgi	1,060	13,915	2,809	11,106	0	11,106	2,920	8,186	3,185	**5,001**
29	77650	Solenzara	979	13,996	4,986	9,010	0	9,010	3,248	5,762	2,895	**2,867**
30	77610	Campo dell Oro	909	14,066	4,923	9,143	0	9,143	3,668	5,475	2,724	**2,751**
31	77800	Sud Corse	928	14,047	4,093	9,954	0	9,954	3,664	6,290	2,437	**3,853**
32	165200	Alguero	1,071	13,904	4,010	9,894	0	9,894	3,452	6,442	2,025	**4,417**
33	165460	Decimomannu	962	14,013	3,391	10,622	0	10,622	3,400	7,222	2,830	**4,392**
34	165600	Elmas	962	14,013	3,391	10,622	0	10,622	3,400	7,222	2,811	**4,411**
35	607150	Carthage	812	14,163	4,370	9,793	5,653	4,140	1,418	2,722	792	**1,930**
36	603600	Annaba	764	14,211	3,003	11,208	8,136	3,072	1,465	1,607	653	**954**
37	604020	Soummam	712	14,263	1,899	12,364	9,675	2,689	1,314	1,375	544	**831**
38	603900	Houari Boumediene	723	14,252	2,401	11,851	8,631	3,220	1,641	1,579	711	**868**
39	604900	Es Senia	817	14,158	1,920	12,238	8,957	3,281	1,346	1,935	638	**1,297**
		**Regional**	891	14,084	3,145	10,939	1,695	9,244	3,323	5,921	2,378	**3,544**
		**Spain**	757	14,218	2,682	11,563	850	10,686	3,814	6,872	2,582	**4,290**
		**France**	916	14,059	4,256	9,804	1,967	7,837	3,481	4,356	2,116	**2,240**
		**Italy**	1,331	13,644	2,645	10,999	1,006	9,992	3,443	6,559	3,074	**3,485**
		**North Africa**	766	14,209	2,719	11,491	8,210	3,280	1,437	1,844	668	**1,176**
		**Balearic Islands**	661	14,314	3,023	11,291	0	11,291	3,724	7,568	2,572	**4,995**
		**Eastern Islands**	993	13,982	3,785	10,197	0	10,197	3,396	6,800	2,770	**4,030**

Table S3. Annual and seasonal trends in the mean SB (a) speed (m s^{-1} $decade^{-1}$) and (b) occurrence (in days $decade^{-1}$) across the Western Mediterranean basin and each coastal region for 1981–2021. Statistically significant trends are defined with an asterisk. NA: North Africa, BI: Balearic Islands, and EI: Eastern Islands.

**(a)**	**Annual**	**Winter (DJF)**	**Spring (MAM)**	**Summer (JJA)**	**Autumn (SON)**
Regional	-0.095*	-0.032	-0.129*	-0.163*	-0.057*
Spain	-0.136*	-0.044*	-0.242*	-0.170*	-0.090*
France	-0.116*	-0.094*	-0.174*	-0.067*	-0.131*
Italy	-0.031	0.088	-0.125*	-0.102*	0.013
NA	-0.069	0.059	-0.132	-0.221*	0.002
BI	-0.138*	-0.156*	-0.168*	-0.139*	-0.091*
EI	-0.065*	-0.070	-0.091*	-0.064*	-0.033
**(b)**	**Annual**	**Winter (DJF)**	**Spring (MAM)**	**Summer (JJA)**	**Autumn (SON)**
Regional	0.62	0.55	0.61	0.05	-0.46
Spain	1.57	1.19	0.74	-0.09	-0.04
France	0.93	-0.36	0.85	0.51	-0.02
Italy	-1.02	0.09	0.04	0.41	-1.38
NA	0.16	0.37	0.20	-0.32	0.00
BI	1.52	0.93	1.51	-0.01	-0.69
EI	-0.14	0.40	0.45	-0.00	-0.96

Table S4. Trends of SB speed at station level over the Western Mediterranean basin for 1981–2021. Trends are expressed in m s^{-1} $decade^{-1}$. Statistically significant trends were defined with an asterisk.

	ID	Annual	Winter (DJF)	Spring (MAM)	Summer (JJA)	Autumn (SON)
1	6155A	-0.207*	-0.116	-0.321*	-0.220*	-0.159*
2	6325O	-0.228*	-0.190*	-0.270*	-0.266*	-0.193*
3	7031	-0.150*	-0.105*	-0.199*	-0.149*	-0.157*
4	8019	-0.099*	0.019	-0.214*	-0.134*	-0.086
5	8025	-0.048*	-0.058	-0.063*	-0.017	-0.066*
6	8414A	-0.071*	0.044	-0.191*	-0.079*	-0.056
7	8416Y	-0.026	0.037	-0.075*	-0.042	-0.016
8	8500A	-0.121*	-0.036	-0.195*	-0.187*	-0.070*
9	9981A	-0.156*	-0.107*	-0.283*	-0.161*	-0.067
10	0016A	-0.128*	0.156	-0.320*	-0.295*	0.024
11	76	-0.297*	-0.162*	-0.557*	-0.349*	-0.150*
12	0200E	-0.085	0.034	-0.160*	-0.143*	-0.072
13	B893	-0.218*	-0.293*	-0.252*	-0.198*	-0.106
14	B278	-0.145*	-0.100	-0.180*	-0.140	-0.105
15	B228	-0.035	-0.084	-0.035	-0.009	0.009
16	B954	-0.151*	-0.080	-0.131*	-0.211*	-0.170*
17	77470	-0.024	-0.064	-0.080	0.095	-0.070
18	76430	-0.185*	-0.209*	-0.179*	-0.130*	-0.236*
19	76500	-0.075*	0.102	-0.180*	-0.134*	-0.085
20	76900	-0.081	0.019	-0.312*	-0.071	-0.014
21	161530	-0.183*	-0.210*	-0.163*	-0.151	-0.168*
22	161680	-0.178	0.229	-0.323*	-0.409*	-0.213
23	162420	0.014	0.096	-0.113	-0.001	0.071
24	162450	-0.032	0.082	-0.222*	-0.053	0.057
25	162890	0.060*	0.114*	0.044	-0.012	0.085
26	163100	0.002	0.037	-0.008	-0.038	0.036
27	164050	-0.020	-0.165	-0.048	0.079	0.055
28	164290	-0.042	-0.098	-0.031	-0.004	-0.041
29	77650	-0.076*	-0.038	-0.151*	-0.069*	-0.051
30	77610	-0.024	-0.042	-0.044	0.027	-0.061*
31	77800	-0.185*	-0.290*	-0.296*	-0.096*	-0.085
32	165200	0.004	-0.104	0.090	0.017	0.010
33	165460	-0.148*	-0.026	-0.153*	-0.224*	-0.115
34	165600	-0.058	0.211	-0.090	-0.242*	-0.024
35	607150	-0.046	0.099	-0.106	-0.221*	0.018
36	603600	0.058	0.207*	-0.233*	-0.427*	0.020
37	604020	-0.071*	-0.043	-0.142*	-0.025	-0.080
38	603900	-0.059	0.066	-0.103	-0.349	-0.075
39	604900	-0.015	-0.055	0.094	0.035	0.126

Table S5. As Table S4 but for SB occurrence. Trends are expressed in days decade^{-1}.

	ID	Annual	Winter (DJF)	Spring (MAM)	Summer (JJA)	Autumn (SON)
1	6155A	1.97	0.41	0.57	0.90	0.04
2	6325O	-2.18	1.99	-0.32	-2.66*	-0.90
3	7031	2.45	1.23	0.89	0.26	0.36
4	8019	1.95	1.57	0.84	-0.33	0.13
5	8025	2.09	1.40	0.88	-0.02	0.11
6	8414A	1.36	1.20	0.55	0.30	-0.30
7	8416Y	1.37	1.33	0.56	0.20	-0.30
8	8500A	1.80	2.13	0.15	0.08	-0.16
9	9981A	2.83*	0.98	0.81	0.82	0.27
10	0016A	1.89	0.83	1.12	-0.61	0.62
11	76	1.72	0.43	1.75	-0.23	-0.13
12	0200E	1.60	0.73	1.02	0.18	-0.24
13	B893	2.81	0.97	1.91	0.54	-0.50
14	B278	1.05	0.75	1.30	0.04	-0.85
15	B228	1.05	0.75	1.30	0.04	-0.85
16	B954	1.18	1.26	1.53	-0.67	-0.55
17	77470	1.52	-0.16	0.62	0.80	0.19
18	76430	1.57	-0.16	0.97	0.90	-0.04
19	76500	1.65	-0.29	0.93	1.22	-0.16
20	76900	0.13	-0.92	1.23	0.09	-0.15
21	161530	-0.25	-0.29	0.52	-0.45	0.05
22	161680	-0.46	0.35	0.37	-0.10	-0.98
23	162420	-2.85	-0.54	-0.01	0.05	-2.12*
24	162450	-2.25	-0.44	-0.05	0.21	-1.76
25	162890	0.14	0.79	-0.35	0.89	-1.05
26	163100	0.31	0.30	0.25	1.00	-0.98
27	164050	-3.12	0.67	-0.69	-0.99	-2.12*
28	164290	-2.70	0.69	-1.02	-0.65	-1.55*
29	77650	0.20	0.37	0.88	0.11	-1.17
30	77610	1.80	-0.03	1.17	1.11	-0.43
31	77800	0.89	0.04	1.22	0.66	-0.98
32	165200	0.61	0.05	1.12	0.23	-0.69
33	165460	0.84	0.75	0.50	-0.06	-0.37
34	165600	0.33	0.68	0.41	-0.40	-0.37
35	607150	-1.73	0.09	-0.45	-0.77	-0.52
36	603600	-0.03	0.44	0.31	-0.53	-0.21
37	604020	0.97	0.33	0.50	-0.10	0.44
38	603900	0.23	0.47	0.26	-0.39*	-0.02
39	604900	1.34	0.50	0.37	0.18	0.35
